# Supplementary material for: Strained few-layer MoS2 with atomic copper and selectively exposed in-plane sulfur vacancies for CO2 hydrogenation to methanol
Source: Nat Commun. 2023 Sep 21;14:5872. doi: 10.1038/s41467-023-41362-y (PMC10514200; doi:10.1038/s41467-023-41362-y)
Supplement: Supplementary file 1 — Supplementary Information [file 41467_2023_41362_MOESM1_ESM.pdf]

Supplementary Information

Strained Few-Layer MoS_2 with Atomic Cu and Selectively Exposed In-plane Sulfur Vacancies for CO_2 Hydrogenation to Methanol

Shenghui Zhou[1,2], Wenrui Ma[1], Uzma Anjum[1], Mohammadreza Kosari[1,3], Shibo Xi[3], Sergey M. Kozlov*,[1], Hua Chun Zeng*,[1,2]

[1] *Department of Chemical and Biomolecular Engineering, College of Design and Engineering, National University of Singapore, Singapore 119260*

[2] *The Cambridge Centre for Advanced Research and Education in Singapore, 1 CREATE Way, Singapore, 138602 Singapore*

[3] *Institute of Sustainability for Chemicals, Energy and Environment (ISCE^2), Agency for Science, Technology and Research (A*STAR), 1 Pesek Road, Jurong Island, 627833 Singapore*

*Emails: sergey.kozlov@nus.edu.sg, chezhc@nus.edu.sg

Table of Contents

1. Supplementary Methods Page S-2 to S-4
2. Supplementary Fig. 1 to Fig. 37 Pages S-5 to S-29
3. Supplementary Table 1 to Table 9 Pages S-30 to S-35
4. Supplementary References Page S-35

1. Supplementary Methods

Chemicals and Materials

Methanol (analytical grade), ethanol (analytical grade) and acetone (analytical grade) were from VWR. Ammonium heptamolybdate tetrahydrate ($(NH_4)_6Mo_7O_{24}\cdot 4H_2O$, AMT, >99%), polyvinylpyrrolidone (PVP, average molecular weight 40000), cetyltrimethylammonium chloride (CTAC, 25 wt% in H_2O), tetraethyl orthosilicate (TEOS, > 99%), copper acetate monohydrate ($Cu(OAc)_2\cdot H_2O$, > 99%), 2-methylimidazole (> 99%), thioacetamide (TAA, > 99%), thiourea (> 99%) and commercial molybdenum disulfide powder (MoS_2-Com, 99%, < 2 µm) were from Sigma-Aldrich. Deionized water was collected through the Elga MicroMeg purified water system.

Syntheses of MoS_2-NPs and MoS_2-HT

MoS_2 nanoparticles (MoS_2-NPs) were obtained from sulfidation of MoO_2 cores. Briefly, 50 mg of MoO_2 cores was dispersed in 40 mL of water with 20 min of sonication, followed by addition of 300 mg of TAA. The solution was transferred to a 50 mL Teflon-lined stainless-steel autoclave container and hydrothermally treated at 200 °C for 24 h. Subsequently, the autoclave was cooled in ambient air, and the resulting black precipitate was collected by centrifugation, washed with ethanol, and dried at 80 °C overnight in an electric oven.

MoS_2-HT was synthesized by traditional hydrothermal methods. Briefly, 340 mg of AMT and 1390 mg of TAA were dissolved in 50 mL of deionized water to form a homogeneous transparent solution. After that, this solution was sealed into a 100 mL Teflon-lined stainless-steel autoclave. The autoclave was put into an oven and maintained at 200 °C for a period of 24 h. The black precipitate was harvested by centrifugation, and washed with deionized water and ethanol several times, and dried at 50 °C under vacuum. The obtained sample was denoted as MoS_2-HT in this work.

Synthesis of pure mesoporous silica spheres (*m*SiO_2)

Pure mesoporous silica spheres (*m*SiO_2) could also be synthesized using the same method of MoO_2@SiO_2 except without the addition of MoO_2 cores.

Synthesis of Cu@SiO_2

Cu@SiO_2 could also be synthesized using the same method of Cu/MoS_2@SiO_2, using the above-prepared *m*SiO_2 spheres instead of the MoS_2@SiO_2.

Synthesis of MoS_2-R

The SiO_2 shell of MoS_2@SiO_2 can be easily removed with KOH solution. Specifically, 400 mg of the MoS_2@SiO_2 was etched in 200 mL of 2.0 M KOH solution for 12 h. After etching, the MoS_2-R sample was washed with water and ethanol three times and then dried at 50 °C overnight in a vacuum drying oven.

Characterization Methods

Field-emission scanning electron microscopy (FESEM, JEOL-6700F), transmission electron microscopy (TEM, JEM-2010, 200 kV), and high-resolution transmission electron microscopy (HRTEM, JEM-2100F, 200 kV) equipped with EDX analysis were applied to observe morphological features of studied products. X-ray diffraction (XRD, Bruker D8 Advance) equipped with a Cu K_α radiation source were carried out to investigate the crystallographic information of the materials. The molybdenum and copper contents in studied catalysts were determined using inductively coupled plasma optical emission spectrometry (ICP-OES, Optima 7300DV, Perkin Elmer, USA). Specific surface areas and average pore size of samples were measured using N_2 adsorption/desorption isotherms at 77 K (Quantachrome NOVA-3000 system). The surface chemical states of representative catalysts were determined by using an X-ray photoelectron spectroscopy (XPS, AXIS-HSi, Kratos Analytical, Al K_α, hv = 1486.71 eV). The binding energies were corrected against the adventitious carbon C 1s peak at 284.5 eV. The investigation of electron paramagnetic resonance spectroscopy (EPR) was carried out using JES-FA (JEOL) under room temperature with microwave frequency of 9.2 GHz. The sample was loaded in a capillary glass tube and the tube was well-sealed to avoid air exposure. For a fair comparison, the MoS_2 masses of all the samples for the EPR test were set the same (ca. 4 mg). Raman spectra of some investigated samples were recorded on a Raman spectrometer (HORIBA Xplora Plus, 638 nm laser at 2 mW) with an aperture of 50 μm, resolution of 3−5 cm^{-1}, microscope objective of 100×, spectral range of 100−1000 cm^{-1}, acquisition time of 10 s, and 10 coadditions. Temperature programmed desorption of H_2 (H_2-TPD) was performed on a Micromeritics AutoChem II 2920 apparatus. Typically, the catalyst (80 mg) was pretreated at 300 °C for 1 h under 10% H_2/Ar flow (25 mL/min) to activate the catalysts. After cooling to room temperature, a flow rate of 25 mL/min of H_2 was introduced for the H_2 adsorption. Subsequently, the purging was carried out at the same temperature by 25 mL/min of Ar for 30 min. Then the temperature was increased linearly from 30 °C to 700 °C with a rate of 5 $°C\cdot min^{-1}$. The gas component in the effluent was monitored and recorded by TCD.

High-pressure *in situ* DRIFTS Experiments

High-pressure *in situ* Diffuse Reflectance Infrared Fourier Transform Spectroscopy (DRIFTS) experiments for some important catalysts were performed with a Bruker TENSOR II spectrometer equipped with a DRIFTS accessory and a cell (Harrick). Each spectrum was recorded at 4 cm^{-1} resolution and related spectral data were based on the average of 64 scans. The as-prepared sample was then sealed inside a high-pressure DRIFTS cell accessory with temperature control (PIKE Technologies DiffusIR), which is installed onto a FTIR machine (Bruker TENSOR II). The calibration was carried out with a flow of Ar gas for 20 min. The catalyst was *in-situ* pretreated with 30 $mL\cdot min^{-1}$ H_2 at 300 °C for 3 h. The DRIFTS cell was subsequently pressurized using the reaction gas to 30 bar regulated by a digital pressure controller (Bronkhorst EL-PRESS). Then the IR spectra were collected at 30 bar and 240 °C. The constant gas flow rate is 20 STP

mL·min^{-1} and the composition of the mixed gas is 72 vol% H_2, 24 vol% CO_2, and 4 vol% N_2.

In situ XANES and EXAFS Experiments

In situ and *ex situ* X-ray absorption spectra (XAS, including both X-ray absorption near-edge structure (XANES) and extended X-ray absorption fine structure (EXAFS)) measurements were conducted using wafer samples (I.D. 10 mm pellets) in a customised high pressure reaction cell equipped with two beryllium windows at the XAFCA beamline of the Singapore Synchrotron Light Source (SSLS). *In situ* XAS measurements were carried out when the samples were sequentially exposed to air (30 °C), He (30 °C), H_2 (300 °C, 2 h), pure CO_2 and reaction gas (H_2:CO_2 = 3:1, 260 °C, 10 bar), respectively. The typical scan time was about 20 min. The XAS data were acquired with energy-calibration by simultaneously recorded transmission spectra of the Cu foil and processed using IFEFFIT software packages.

The Detailed Calculation Procedure for Reaction Parameters

The reaction parameters including CO_2 conversion, product selectivity, methanol space-time yield (STY_{MeOH}), specific MeOH yield were calculated as Eq. (1)-(4):

$$CO_2 \text{ conversion } \% = \frac{n_{CO_2,in} - n_{CO_2,out}}{n_{CO_2,in}} \times 100 \quad (1)$$

$$\text{Product selectivity } \% = \frac{n_{product}}{\sum n_{product}} \times 100 \quad (2)$$

$$STY_{MeOH}\ (g_{MeOH} \cdot g_{MoS_2}^{-1} \cdot h^{-1}) = n_{MeOH} \times \frac{M_{MeOH}}{m_{MoS_2}} \times 60\ (\text{min} \cdot h^{-1}) \times 0.001\ (\text{mol} \cdot \text{mmol}^{-1}) \quad (3)$$

$$\text{Specific MeOH yield } (mol_{MeOH} \cdot mol_{Mo}^{-1} \cdot h^{-1}) = STY_{MeOH} \times M_{MoS_2}/M_{MeOH} \quad (4)$$

where $n_{CO_2,in}$ and $n_{CO_2,out}$ are the amounts of CO_2 (mol) at the inlet and outlet of the reactor, $n_{product}$ is the amount of product (mol) at the outlet of the reactor. m_{MoS_2} is the weight of MoS_2 in the catalyst (g), M_{MoS_2} is the molecular weight of MoS_2 (160.07 g·mol^{-1}) and M_{MeOH} is the molecular weight of methanol (32.04 g·mol^{-1}). In order to check the validity of the above calculation method, the carbon balance was also monitored in every reaction run and it was found to be higher than 98% in all our experiments.

2. Supplementary Fig. 1 to Fig. 37

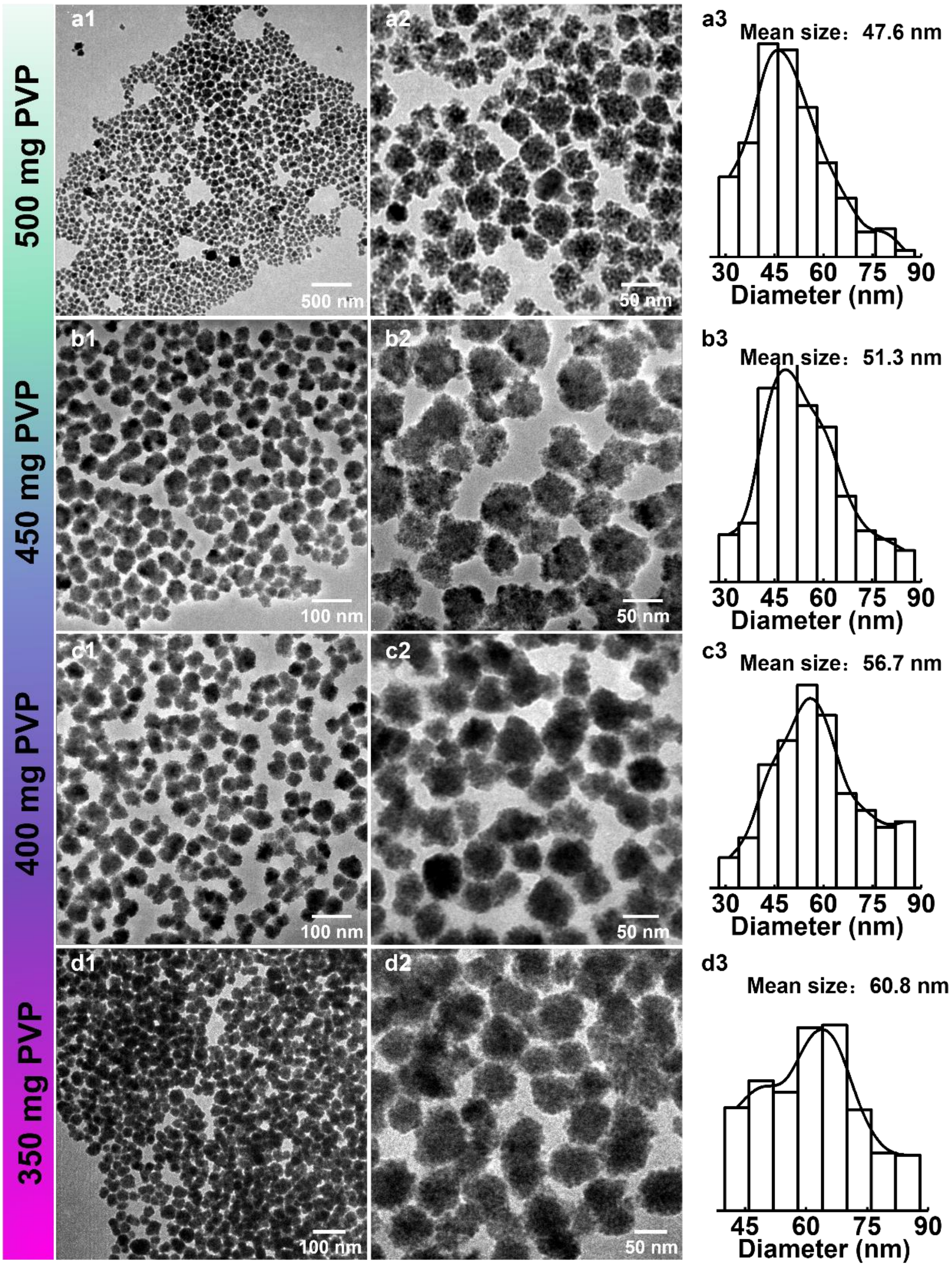

Supplementary Fig. 1. TEM images of MoO_2 obtained with different added PVP amounts. Reaction conditions: 180 °C, 16 h, 150 mg of AMT, 22 mL of water, 10 mL of ethanol. (a1-a2) 500 mg of PVP, (b1-b2) 450 mg of PVP, (c1-c2) 400 mg of PVP, (d1-d2) 350 mg of PVP. (a3-d3) corresponding particle size distribution of MoO_2.

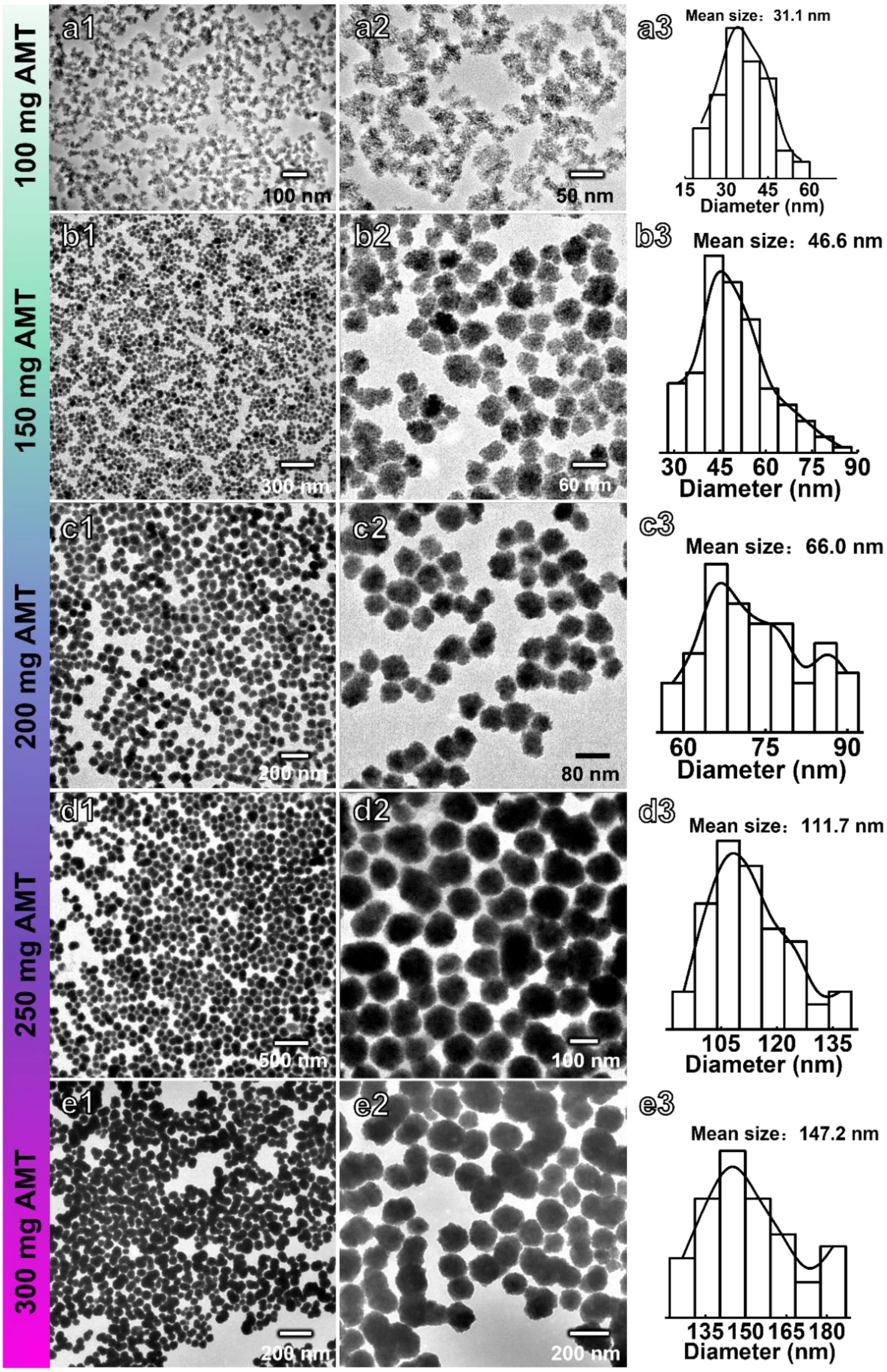

Supplementary Fig. 2. TEM images of MoO_2 obtained with different added AMT amounts. Reaction conditions: 180 °C, 16 h, 500 mg of PVP, 22 mL of water, 10 mL of ethanol. (a1-a2) 100 mg AMT, (b1-b2) 150 mg of AMT, (c1-c2) 200 mg of AMT, (d1-d2) 250 mg of AMT, (e1-e2) 300 mg of AMT. (a3-e3) corresponding particle size distribution of MoO_2.

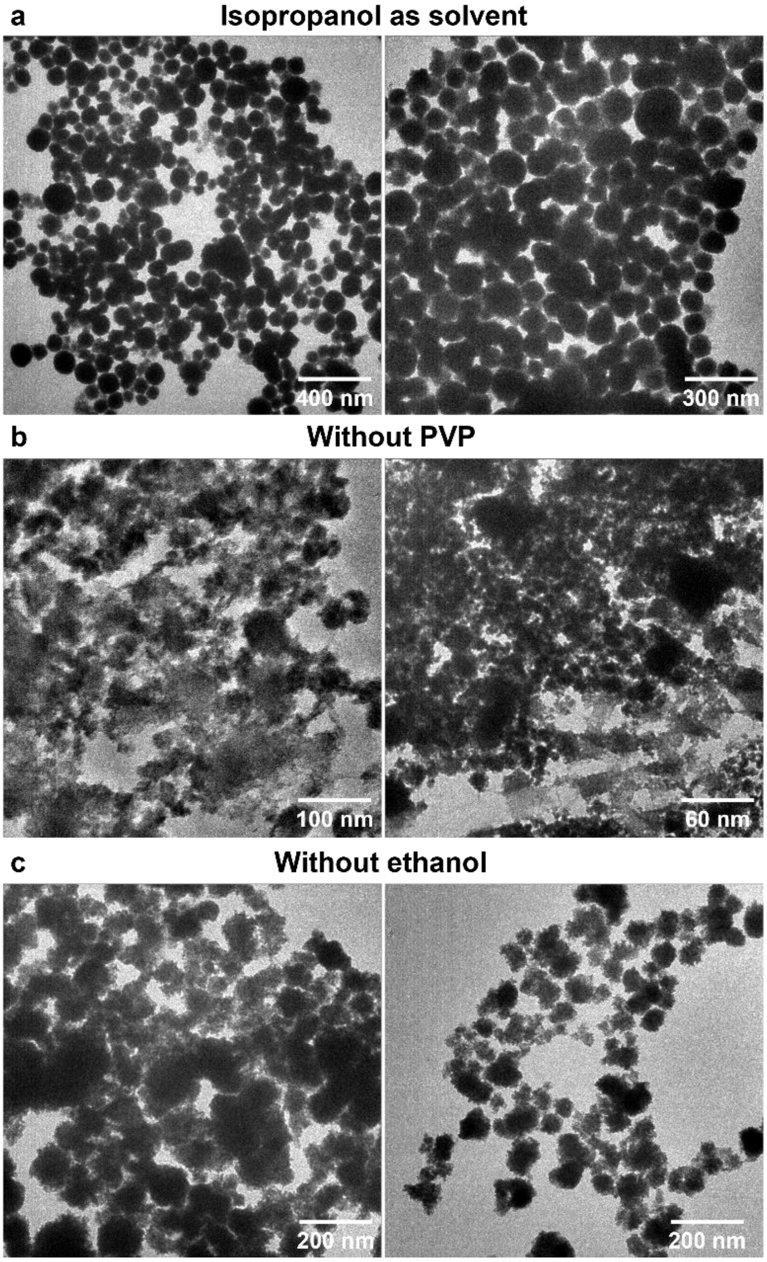

Supplementary Fig. 3. TEM images of MoO_2 obtained with reaction conditions. Standard reaction conditions: 180 °C, 16 h, 500 mg of PVP, 150 mg of AMT, 22 mL of water, 10 mL of ethanol. (a) ethanol was replaced with isopropanol, (b) without PVP, (c) without ethanol.

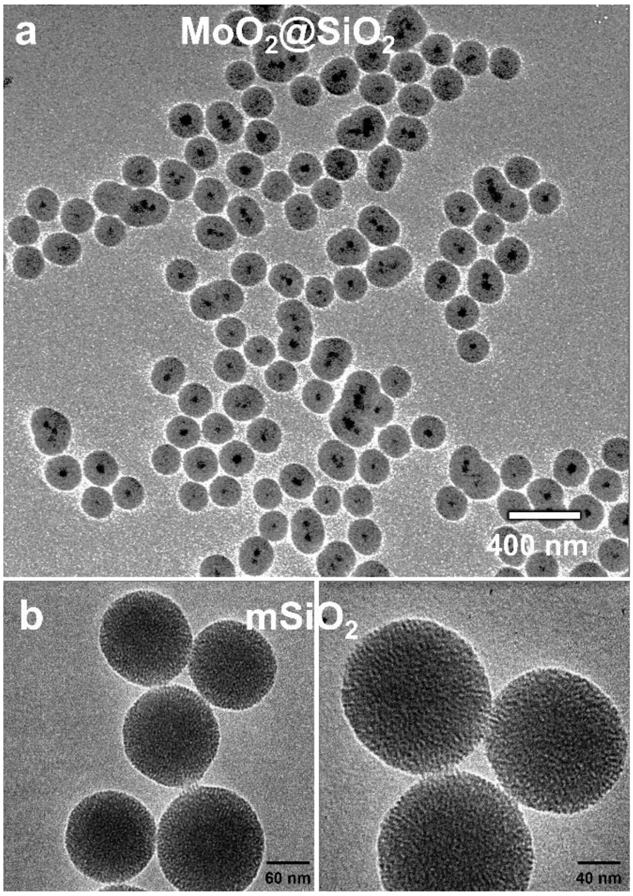

Supplementary Fig. 4. TEM images of (a) $MoO_2@SiO_2$ and (b) mesoporous silica spheres.

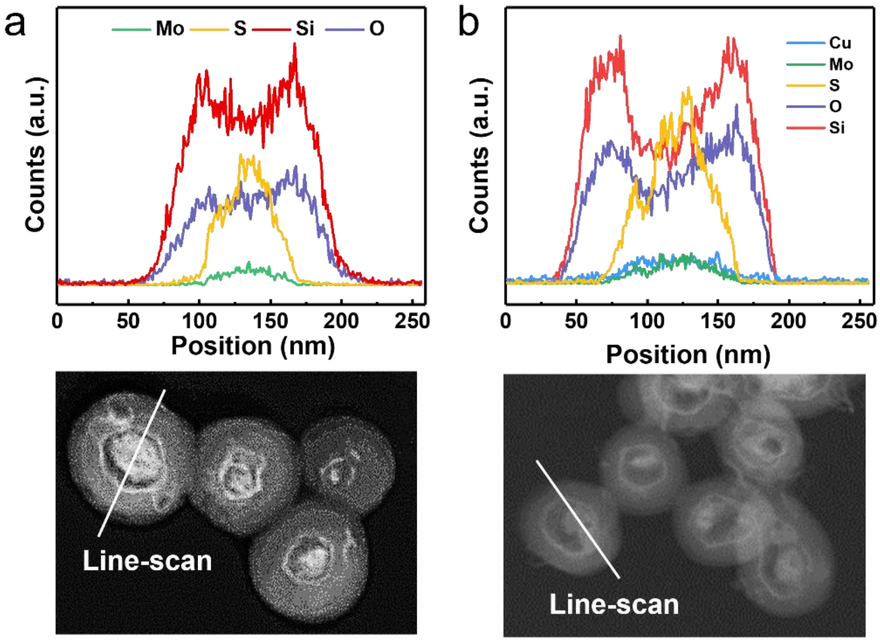

Supplementary Fig. 5. HRTEM-EDX line profiles of (a) $MoS_2@SiO_2$ and (b) $Cu/MoS_2@SiO_2$ along the white line of corresponding image.

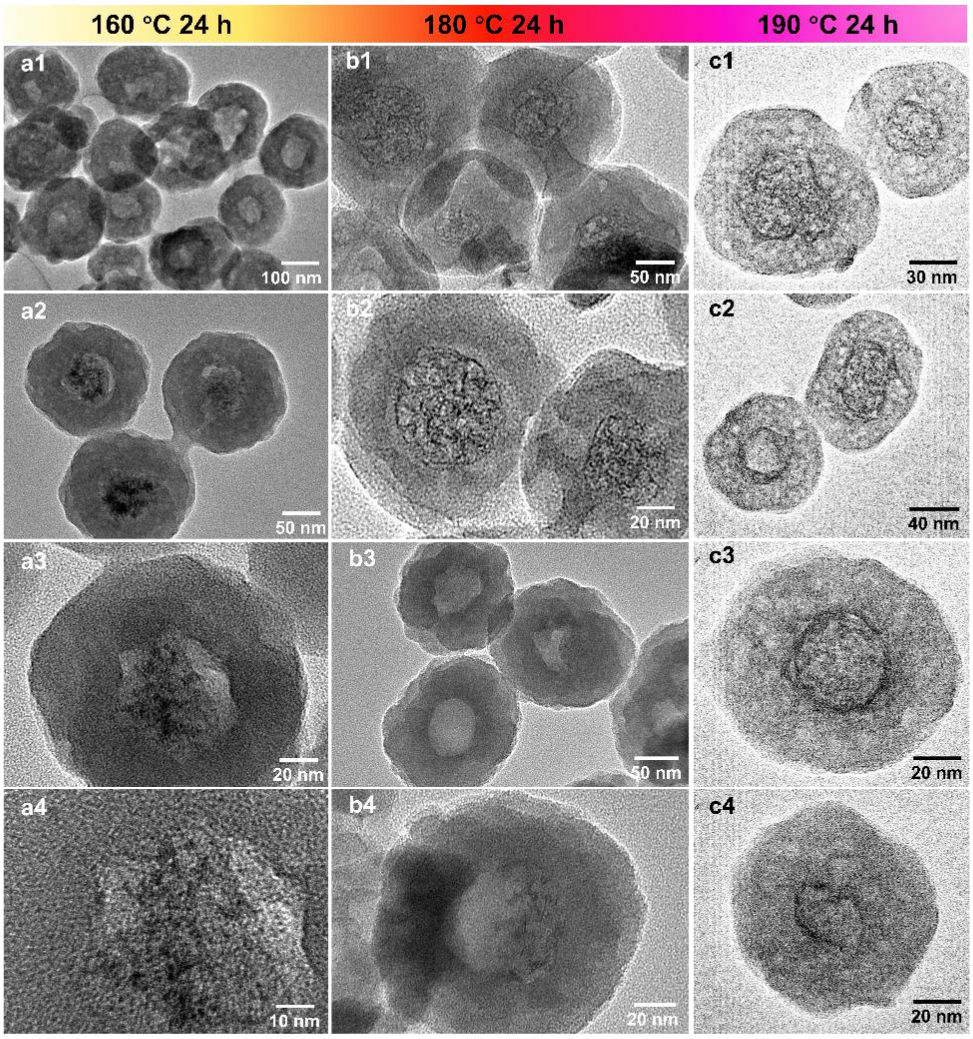

Supplementary Fig. 6. TEM images of MoS_2@SiO_2 obtained at different hydrothermal temperature. Reaction conditions: 24 h, 100 mg of MoO_2@SiO_2, 300 mg of TAA, 40 mL of water. (a1-a3) 160 °C, (b1-b3) 180 °C, (c1-c3) 190 °C.

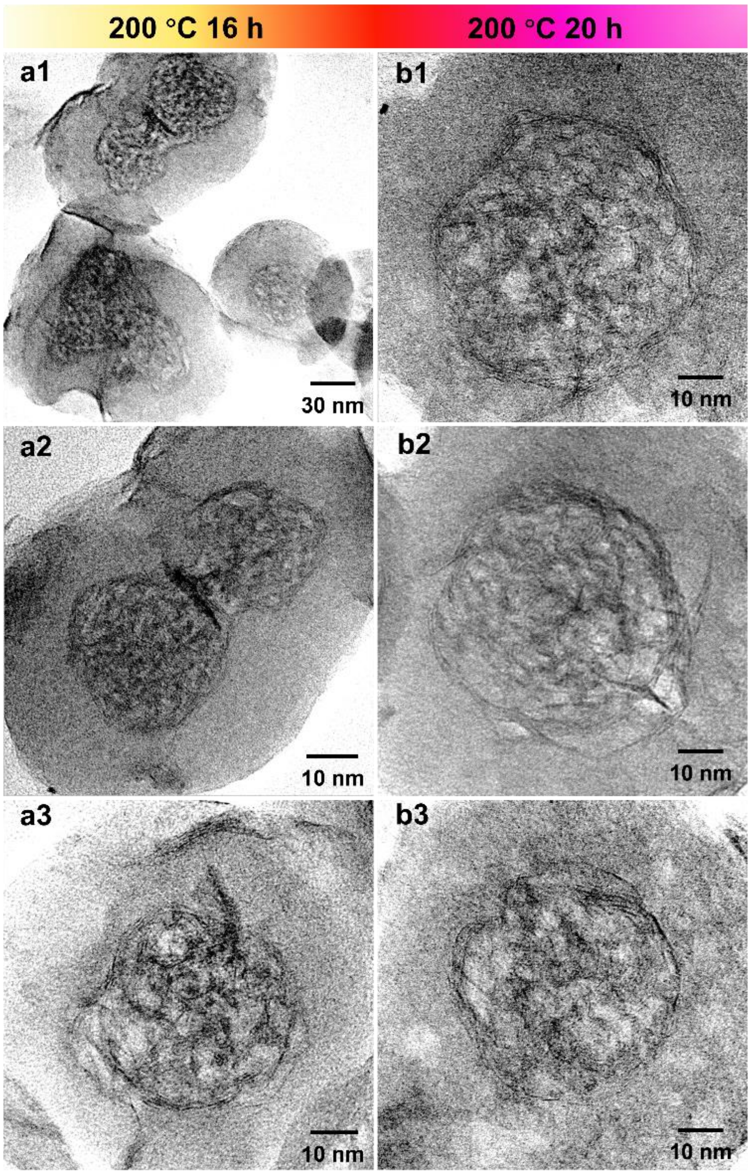

Supplementary Fig. 7. TEM images of $MoS_2@SiO_2$ obtained at different hydrothermal times. Reaction conditions: 200 °C, 100 mg of $MoO_2@SiO_2$, 300 mg of TAA, 40 mL of water. (a1-a3) 16 h, (b1-b3) 20 h.

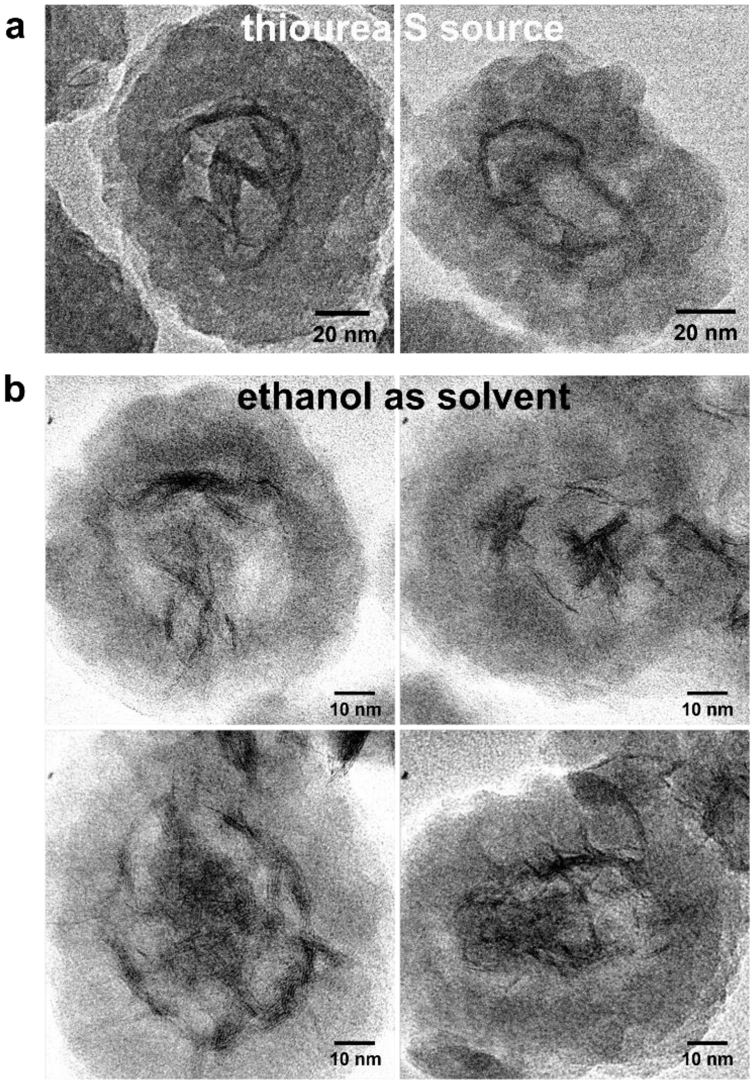

Supplementary Fig. 8. TEM images of MoS_2@SiO_2 obtained with thiourea as S source (a). Reaction conditions: 200 °C, 24 h, 100 mg of MoO_2@SiO_2, 300 mg of thiourea, 40 mL of water. TEM images of MoS_2@SiO_2 in ethanol solvent (b). Reaction conditions: 180 °C, 24 h, 100 mg of MoO_2@SiO_2, 300 mg of TAA, and 35 mL of ethanol.

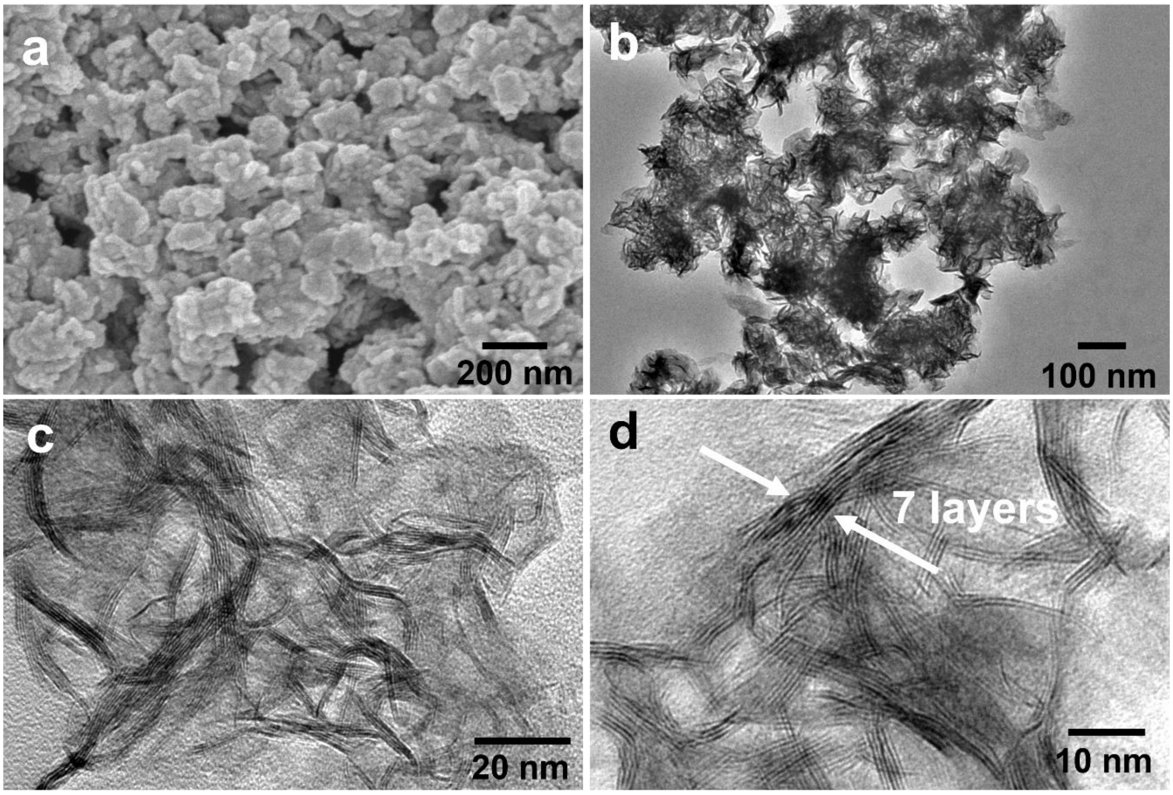

Supplementary Fig. 9. FESEM (a) and TEM images (b-c) of MoS_2-NPs.

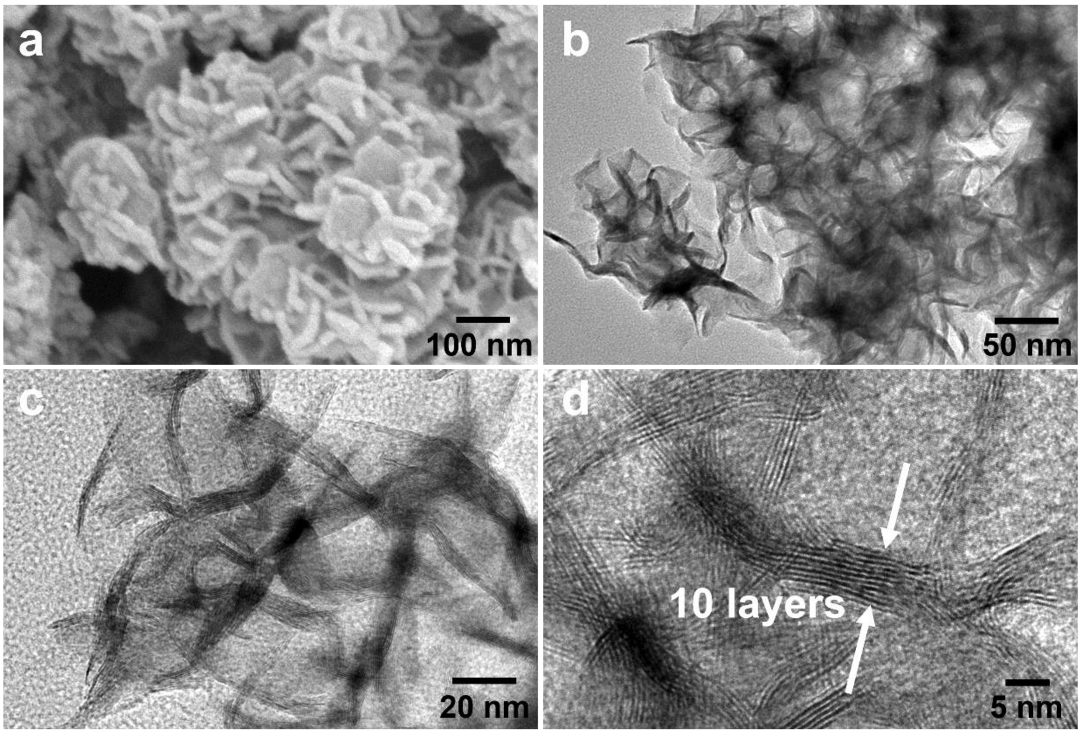

Supplementary Fig. 10. FESEM (a) and TEM images (c-d) of MoS_2-HT.

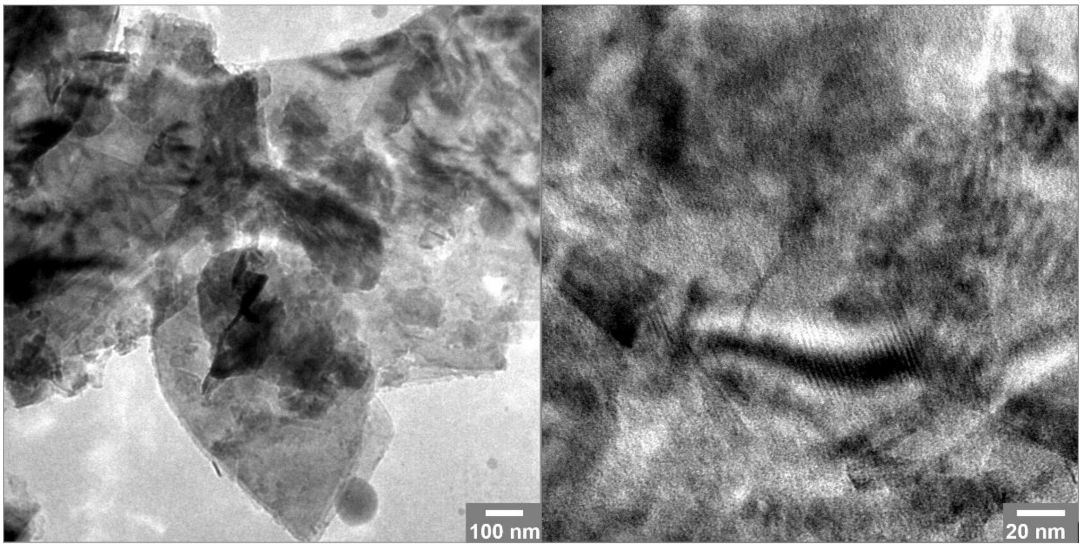

Supplementary Fig. 11. TEM images of commercial MoS_2 (MoS_2-Com) from Sigma-Aldrich.

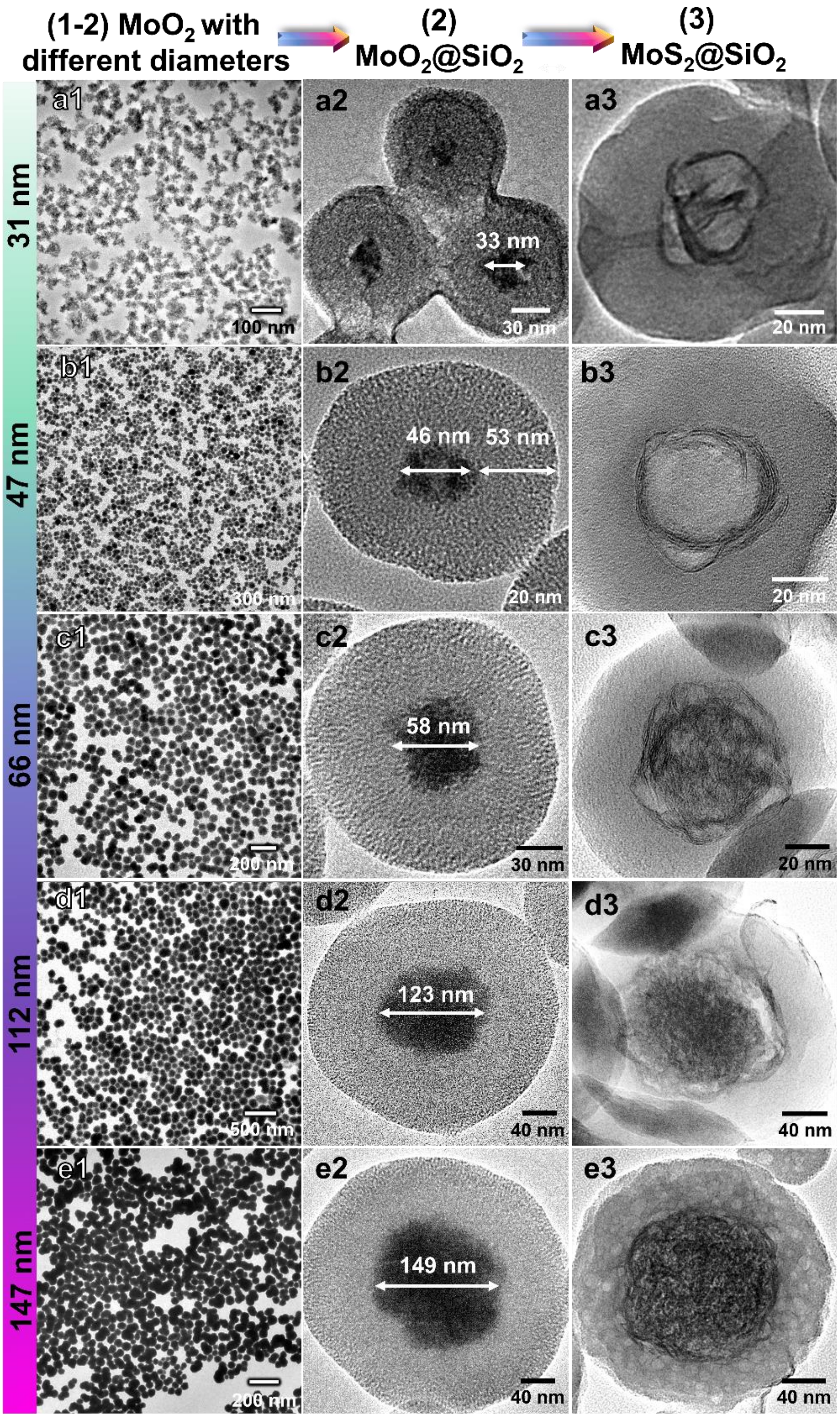

Supplementary Fig. 12. Influence of the MoO_2 diameters on final morphology of $MoS_2@SiO_2$. (a1-e1) MoO_2, (a2-e2) $MoO_2@SiO_2$, and (a3-e3) $MoS_2@SiO_2$.

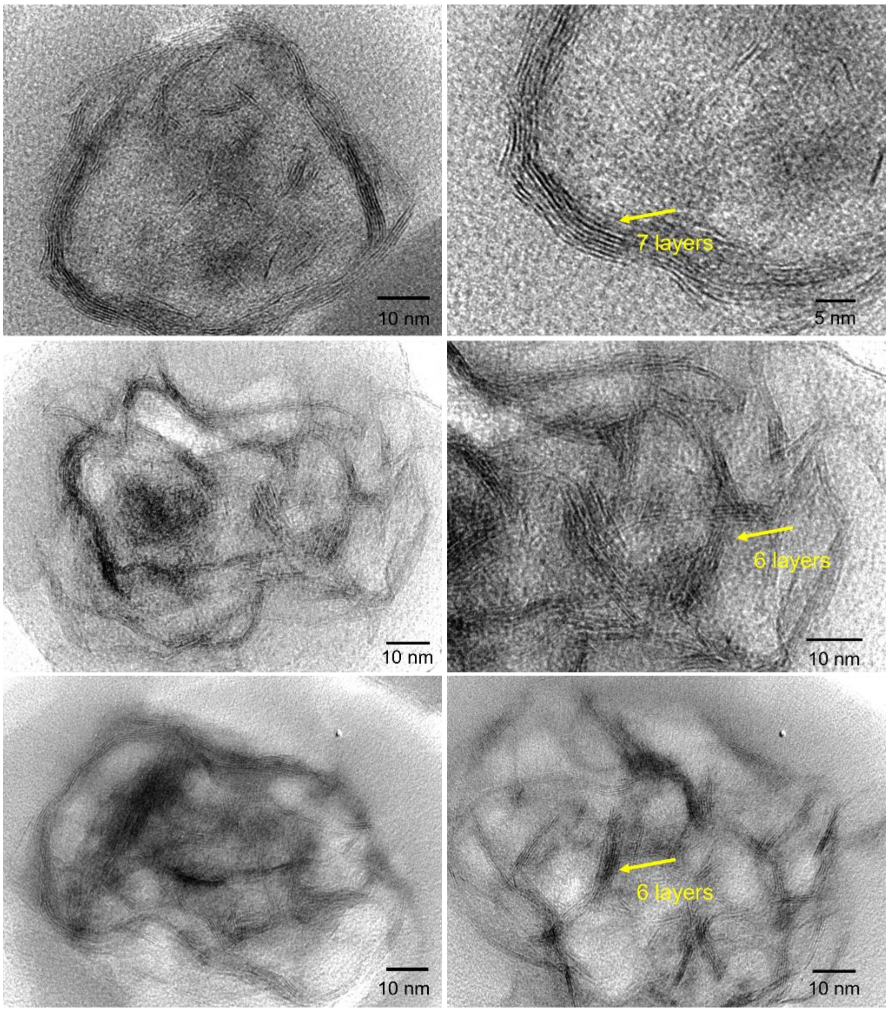

Supplementary Fig. 13. TEM images of MoS_2@SiO_2-66nm (MoO_2 with an average diameter of 66 nm as Mo source). The sulfidation time is 36 h.

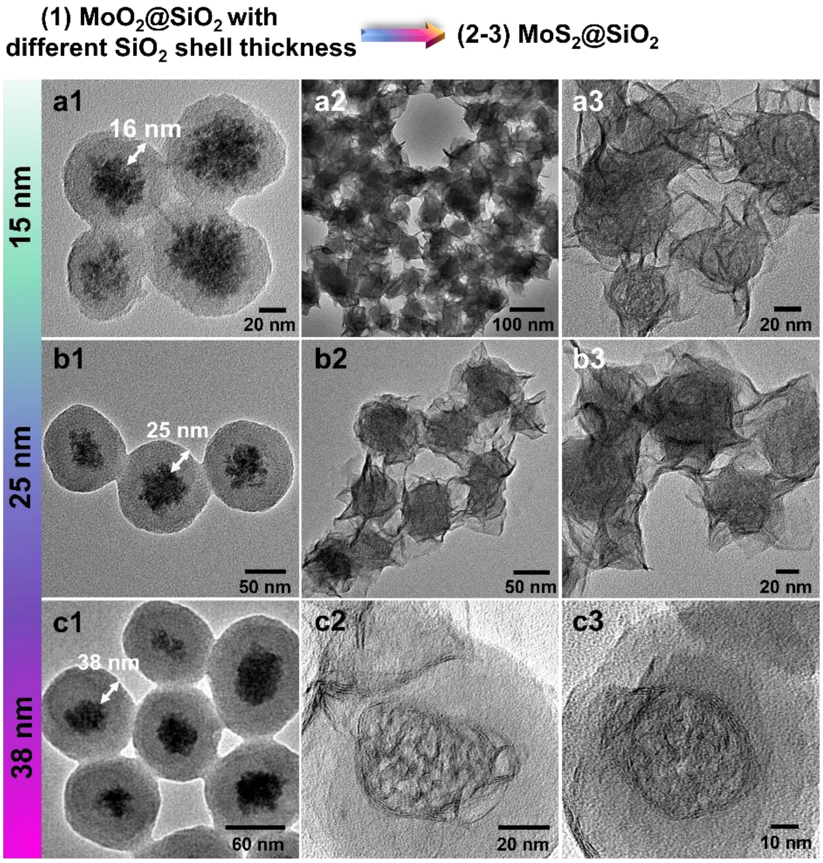

Supplementary Fig. 14. Influence of the SiO_2 shell thickness on final morphology of MoS_2@SiO_2. (a1-c1) MoO_2@SiO_2, and (a2, a3-c2, c3) MoS_2@SiO_2.

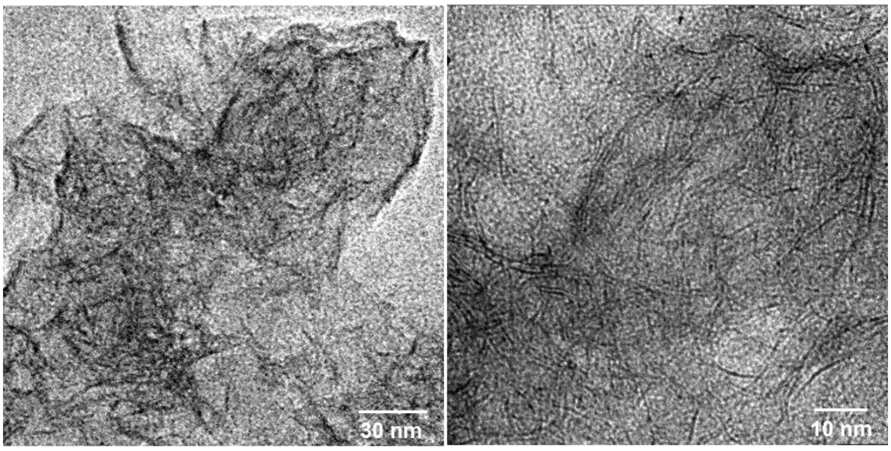

Supplementary Fig. 15. TEM images of MoS_2-R (the SiO_2 shell of MoS_2@SiO_2 were removed).

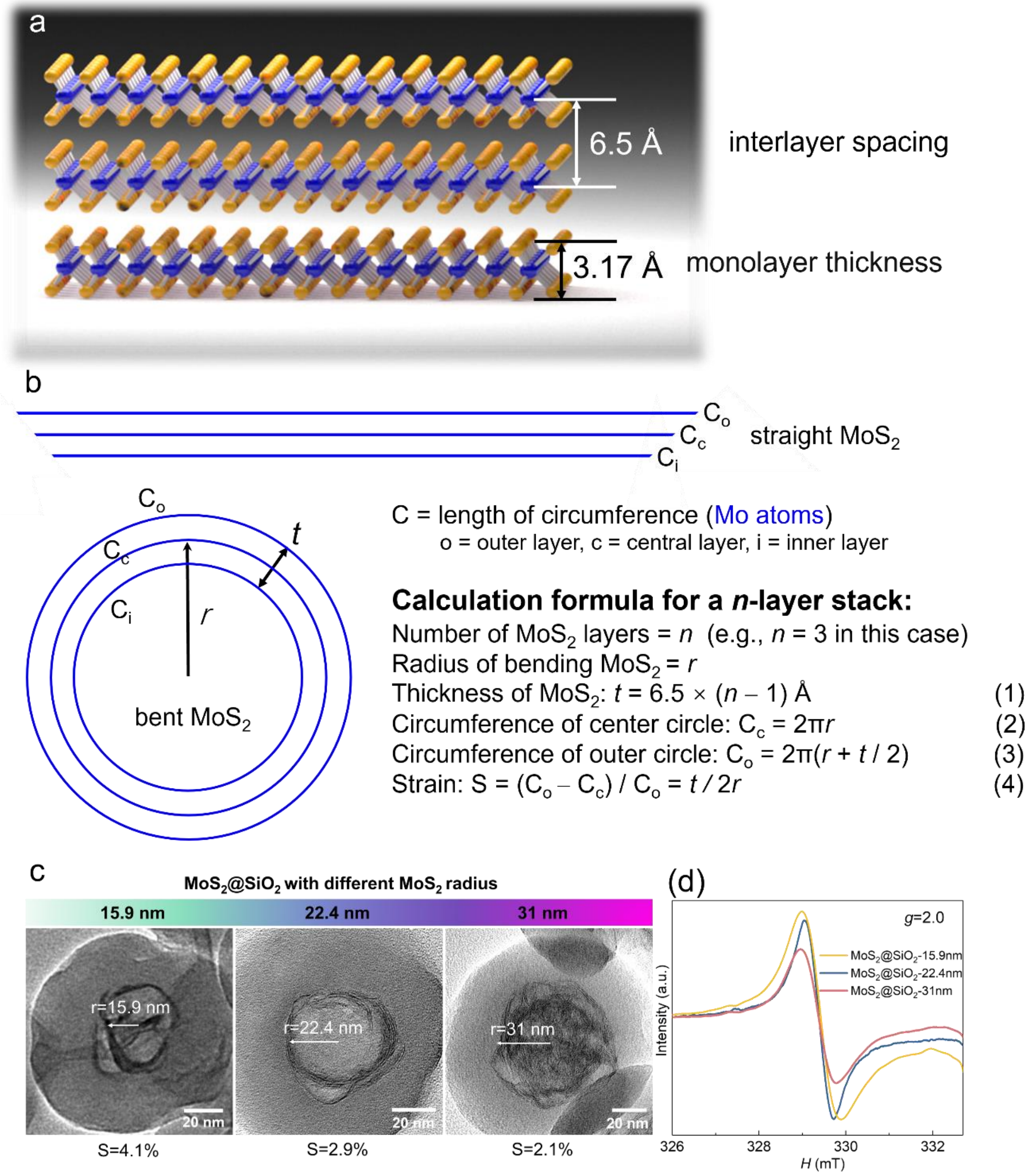

Supplementary Fig. 16. Calculation of in-plane uniaxial strain of MoS_2@SiO_2 with different MoS_2 radius. (a) interlayer spacing of MoS_2 and thickness of monolayer MoS_2, (b) calculation formula of in-plane uniaxial compressive strain (Note: The illustration is focused on three different Mo atom layers only for simplicity; furthermore, a detailed analysis for intralayer tensile strain and compressive strain within a single MoS_2 layer can be found in our DFT calculations), (c) calculation results according to (b), and (d) corresponding EPR spectra of our studied samples.

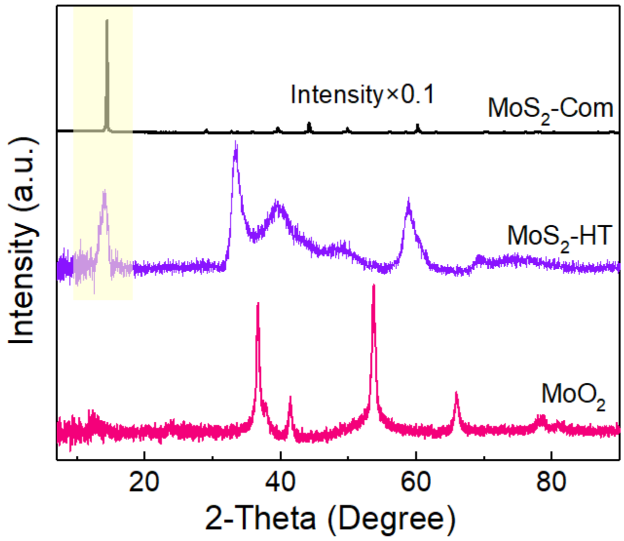

Supplementary Fig. 17. XRD patterns of MoO_2, MoS_2-HT and MoS_2-Com samples.

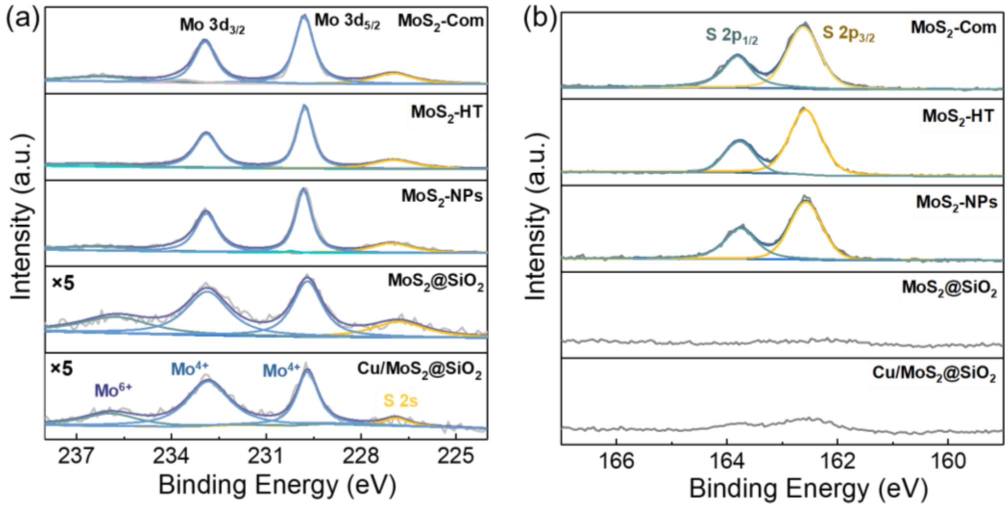

Supplementary Fig. 18. Mo 3*d* high-resolution XPS spectra (a), and S 2*p* high-resolution XPS spectra (b) of various samples studied in this work.

Note: Due to the extremely small size of the synthesised MoO_2 cores and constrained sulfidation process, the MoS_2 formed does not aggregate and MoO_2 cores can be converted into few-layered MoS_2. In contrast, MoS_2 counterparts (MoS_2-HT, MoS_2-NPs, MoS_2-Com) have a multilayer or thick-layered nanosheet structure. It is clear that the highly exposed few-layered MoS_2 of MoS_2@SiO_2 and Cu/MoS_2@SiO_2 are more active for hydrogenation but at the same time more susceptible to be oxidized to high valence. For MoS_2 samples with a thick nanosheet structure (MoS_2-HT, MoS_2-NPs, MoS_2-Com), most of the Mo is located in the middle of the nanosheet layer, which makes it less susceptible to oxidation. Therefore, Mo^{+6} content in MoS_2@SiO_2 and Cu/MoS_2@SiO_2 with few-layer MoS_2 structure is higher when compared to multi-layered samples (MoS_2-HT, MoS_2-NPs, MoS_2-Com).

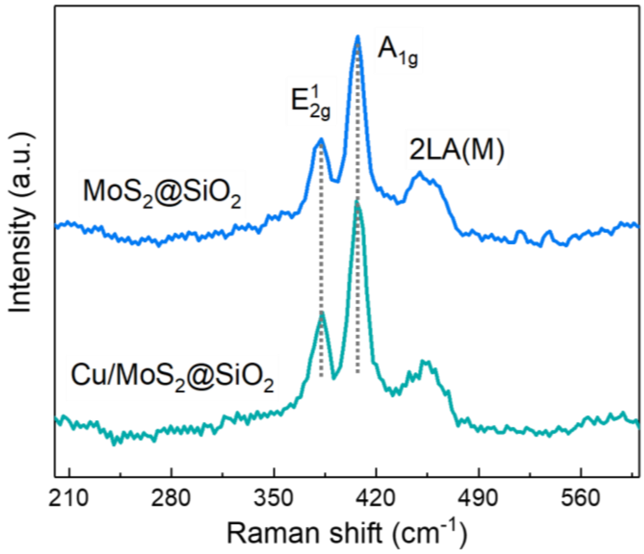

Supplementary Fig. 19. Raman spectra of $MoS_2@SiO_2$ and $Cu/MoS_2@SiO_2$.

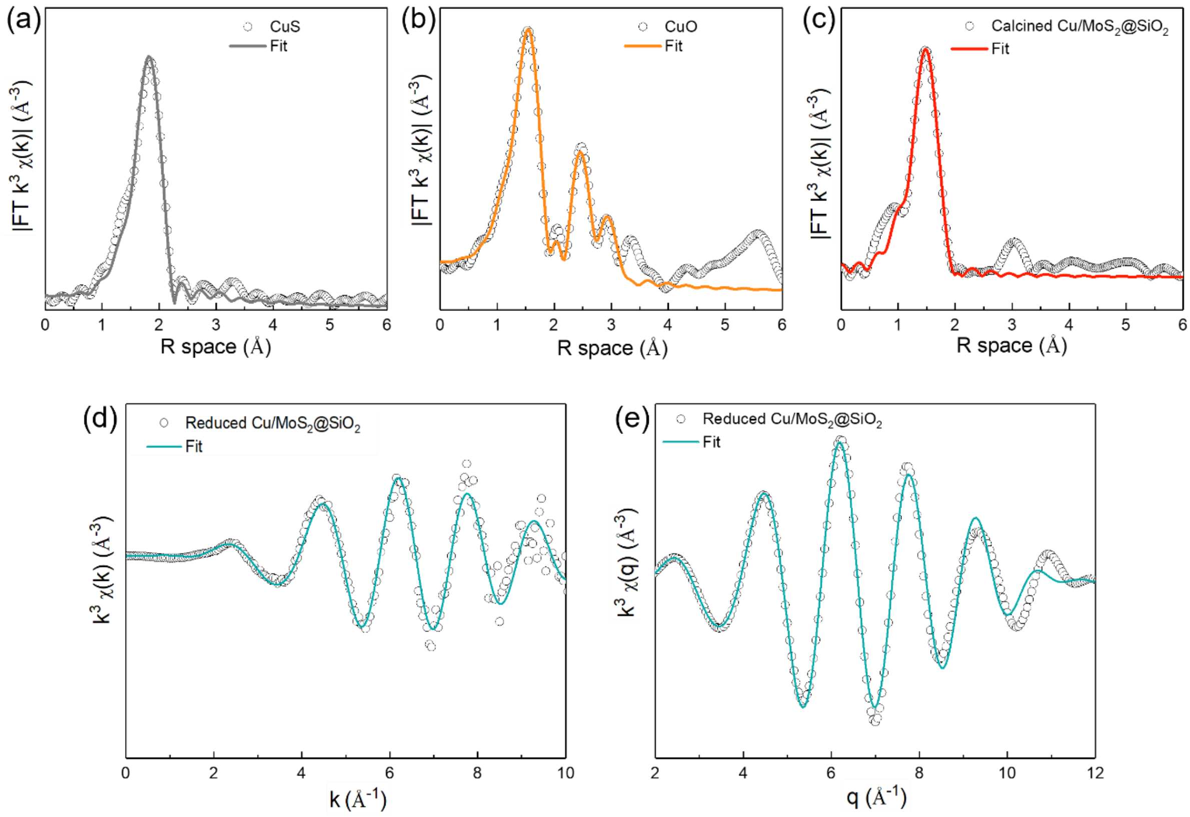

Supplementary Fig. 20. FT-EXAFS fitting curves of CuS (a), CuO (b) and calcined $Cu/MoS_2@SiO_2$ (c) in R space, (d) k space fitting curve and (e) q space fitting curve at Cu K-edge of reduced $Cu/MoS_2@SiO_2$.

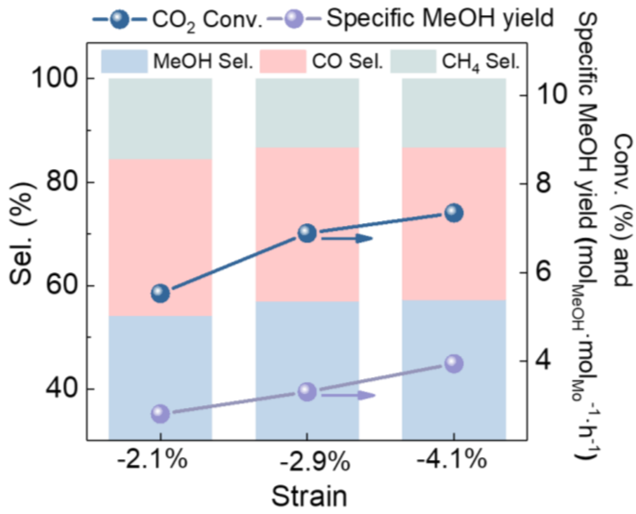

Supplementary Fig. 21. Catalytic performance comparison of $MoS_2@SiO_2$ with different strain. The $MoS_2@SiO_2$ with different strain (–2.1%, –2.9%, –4.1%) were prepared by using MoO_2 with different particle size (31 nm, 47 nm, 66 nm). Reaction condition: 260 °C, 5 MPa, GHSV = 24000 mL·$g_{cat.}^{-1}$·h^{-1}.

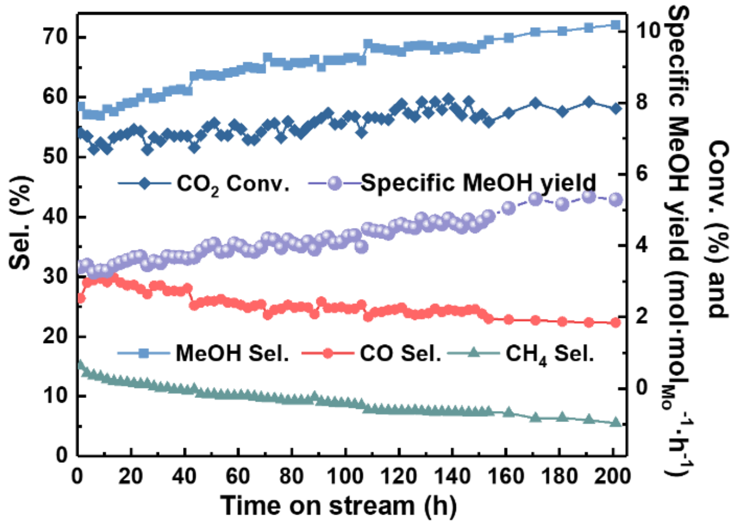

Supplementary Fig. 22. Long-term tests of $MoS_2@SiO_2$. Reaction conditions: 260 °C, 5 MPa, CO_2:H_2 = 1:4, and GHSV = 24000 mL·$g_{cat.}^{-1}$·h^{-1}.

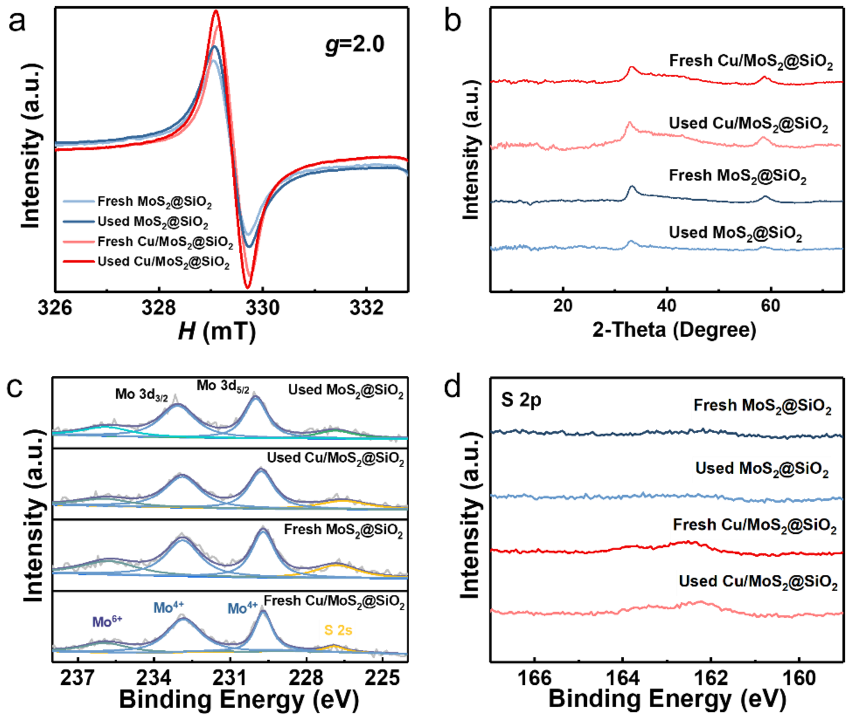

Supplementary Fig. 23. EPR spectra (a), XRD patterns (b), and high-resolution XPS spectra of H_2 pretreated catalysts (i.e., fresh) and used catalysts after long-term stability experiments: Mo 3*d* (c) and S 2*p* (d).

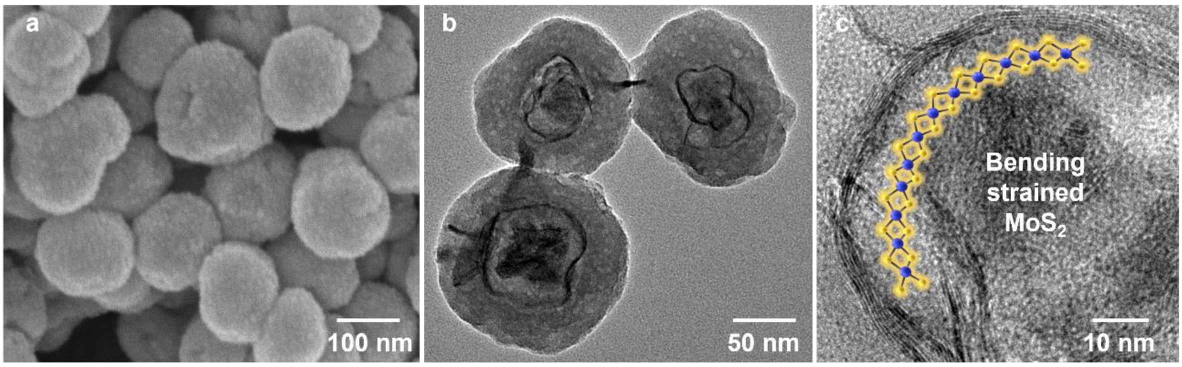

Supplementary Fig. 24. SEM image (a) and TEM images (b, c) of used $MoS_2@SiO_2$ after the long-term stability experiment.

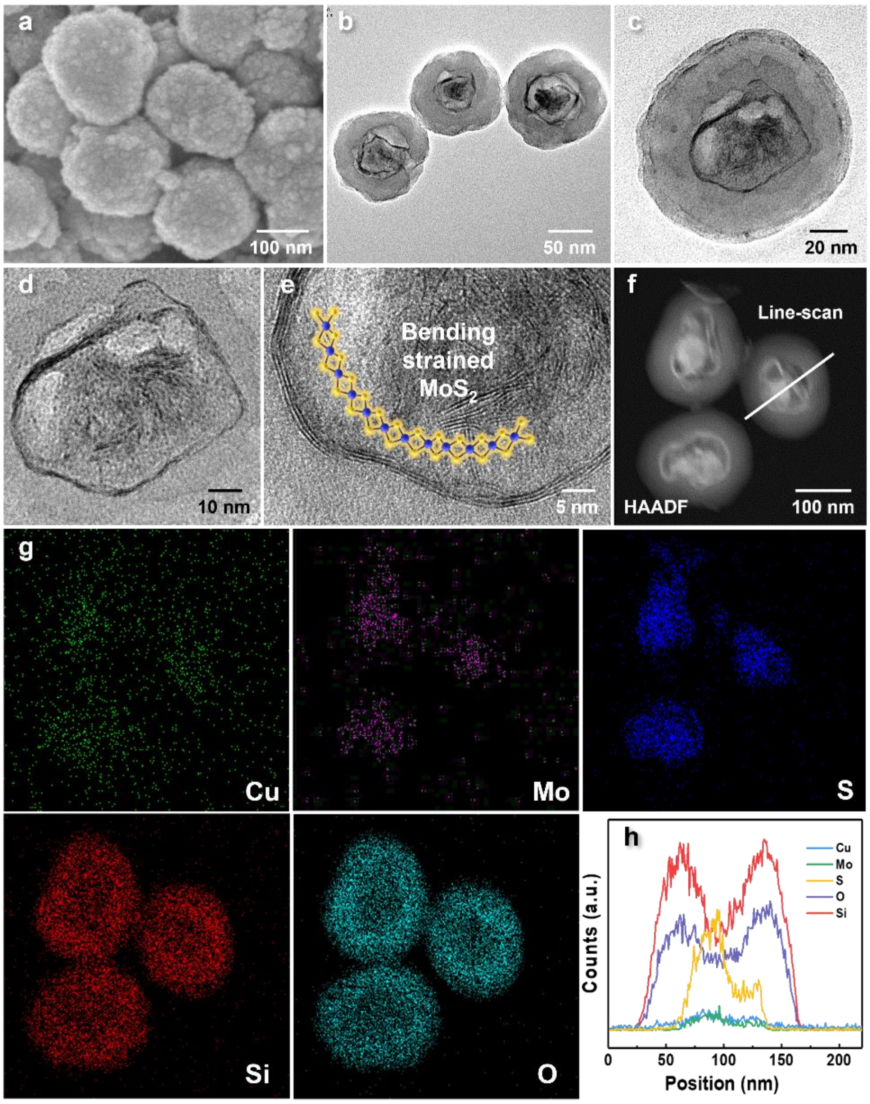

Supplementary Fig. 25. SEM image (a), TEM images (b-e), HAADF-STEM image (f), corresponding elemental mappings (g), and EDX line profiles (h) of used $Cu/MoS_2@SiO_2$ after long-term stability experiment.

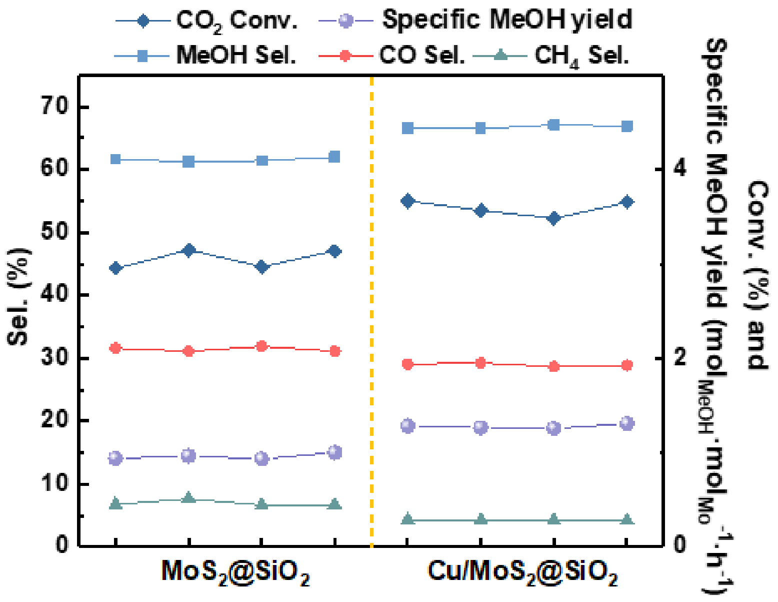

Supplementary Fig. 26. Conversion, selectivity and specific MeOH yield for CO_2 hydrogenation of MoS_2@SiO_2 and Cu/MoS_2@SiO_2 under low pressure and low temperature conditions. Reaction conditions: 230 °C, 2.5 MPa, CO_2:H_2 = 1:4, GHSV = 12000 mL·$g_{cat.}^{-1}$·h^{-1}.

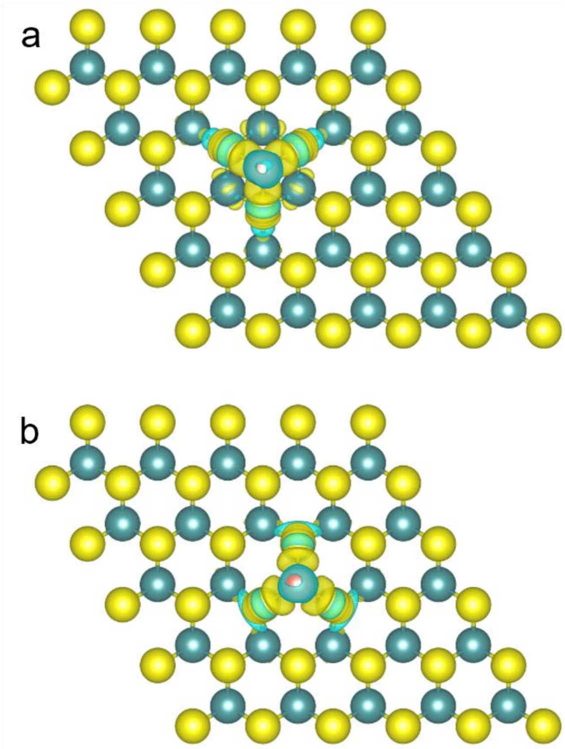

Supplementary Fig. 27. Charge density difference plots of Cu/MoS_2 with Cu at (a) hollow and (b) Mo atop sites. The charge density difference $\Delta\rho$ is defined as $\Delta\rho = \rho_{Cu/MoS_2} - \rho_{MoS_2} - \rho_{Cu}$, where ρ_{Cu/MoS_2}, ρ_{MoS_2}, and ρ_{Cu} are charge densities of Cu-supported MoS_2, pure MoS_2 fixed at the geometry with supported Cu, and single Cu atom. Yellow and cyan represent isosurfaces of electron accumulation and depletion, respectively, plotted with an isovalue of 0.002 e/Å^3.

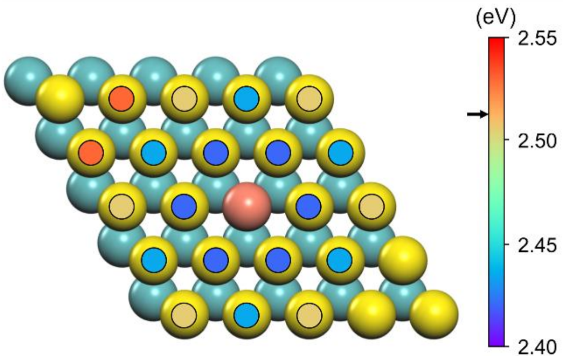

Supplementary Fig. 28. Formation energies of the second S vacancy when a Cu atom is located at the first S vacancy site. The arrow indicates the lowest vacancy formation energy of the second S vacancy in the absence of Cu.

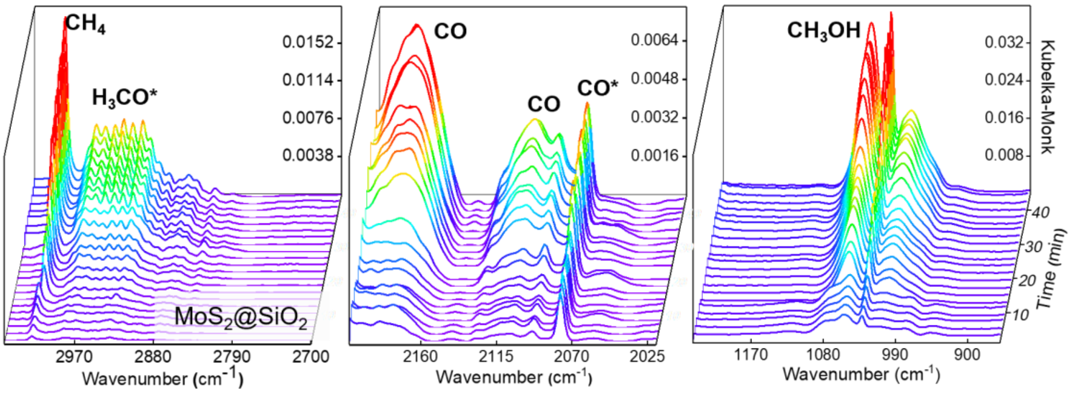

Supplementary Fig. 29. High pressure *in situ* DRIFT spectra of the CO_2 hydrogenation to methanol reaction catalyzed by MoS_2@SiO_2.

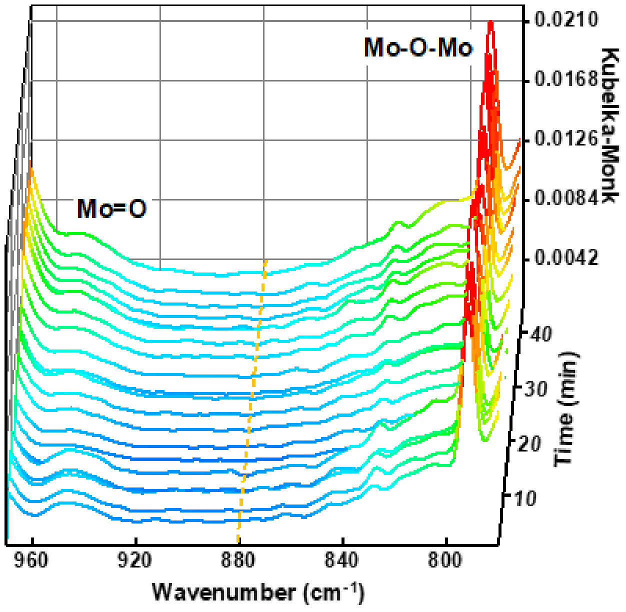

Supplementary Fig. 30. High pressure *in situ* DRIFT spectra of the CO_2 hydrogenation to methanol reaction catalyzed by Cu/MoS_2@SiO_2. Absorbance bands from 770~980 cm^{-1}.

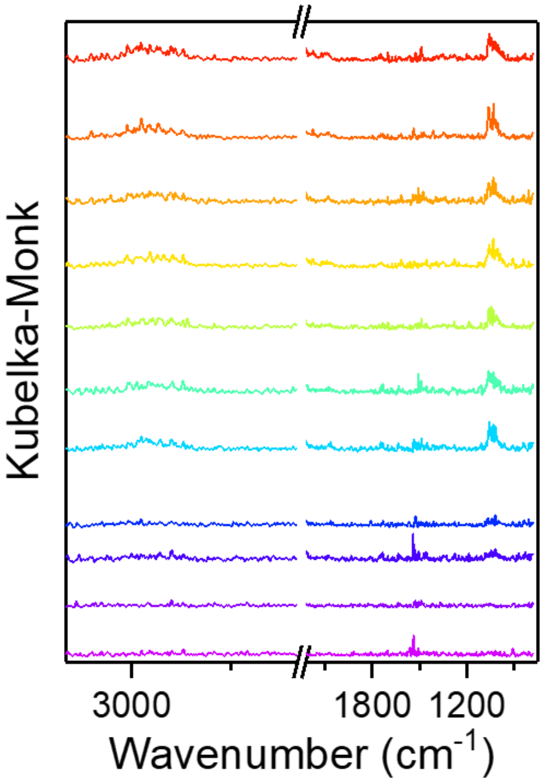

Supplementary Fig. 31. High pressure *in situ* DRIFT spectra of the CO_2 hydrogenation to methanol reaction catalyzed by MoS_2-NPs.

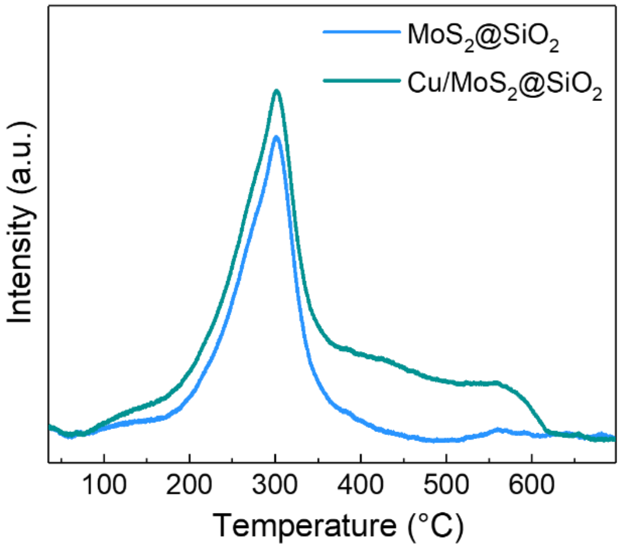

Supplementary Fig. 32. H_2-TPD of MoS_2@SiO_2 and Cu/MoS_2@SiO_2.

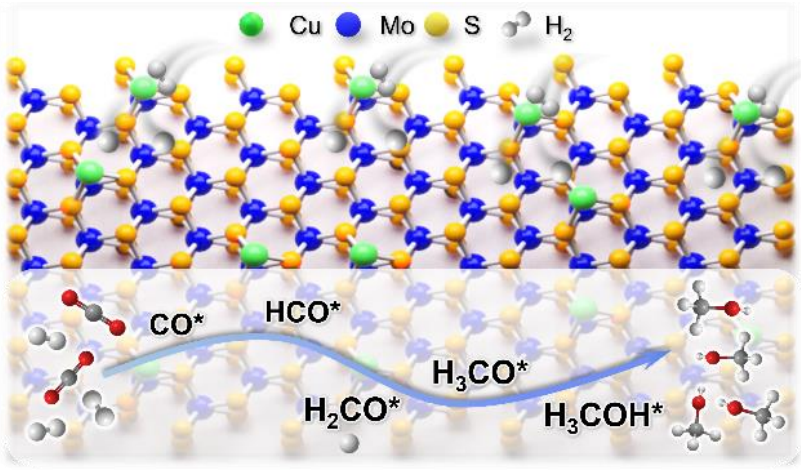

Supplementary Fig. 33. Plausible reaction mechanism for CO_2 hydrogenation over Cu/MoS_2@SiO_2 catalyst.

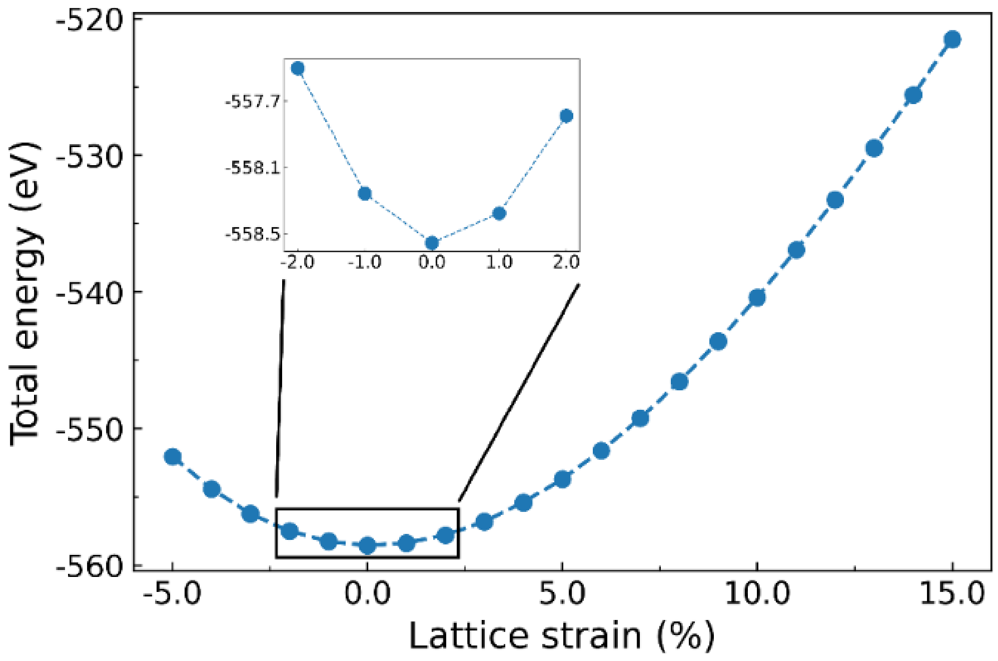

Supplementary Fig. 34. Total energy of the pristine MoS_2 monolayer film at different strain levels.

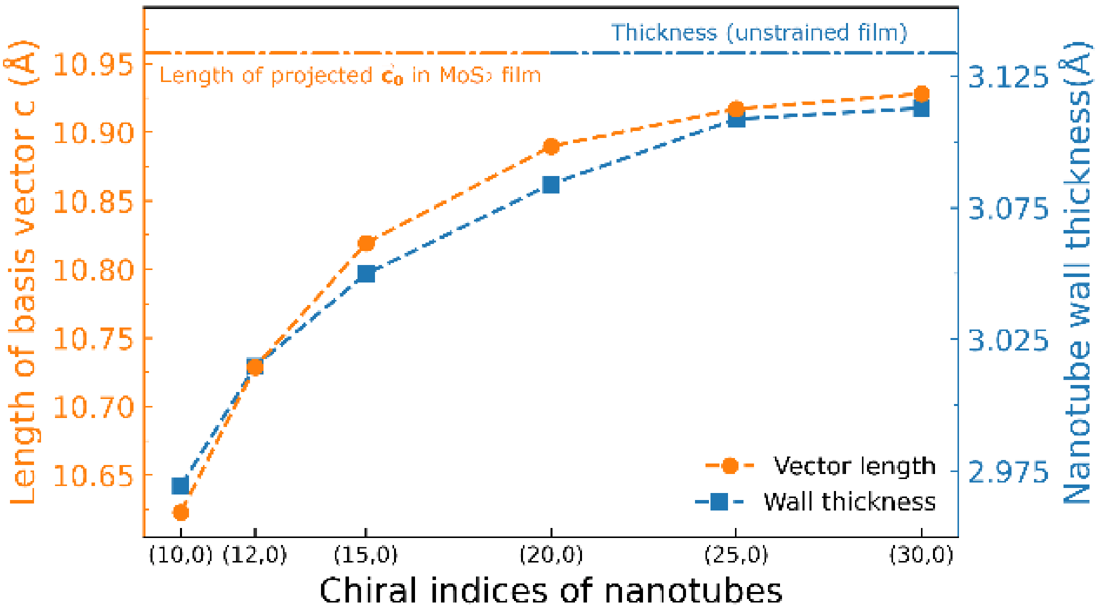

Supplementary Fig. 35. Optimized length of basis vector $\vec{c}$ of nanotubes with different chiral indices and the corresponding wall thickness.

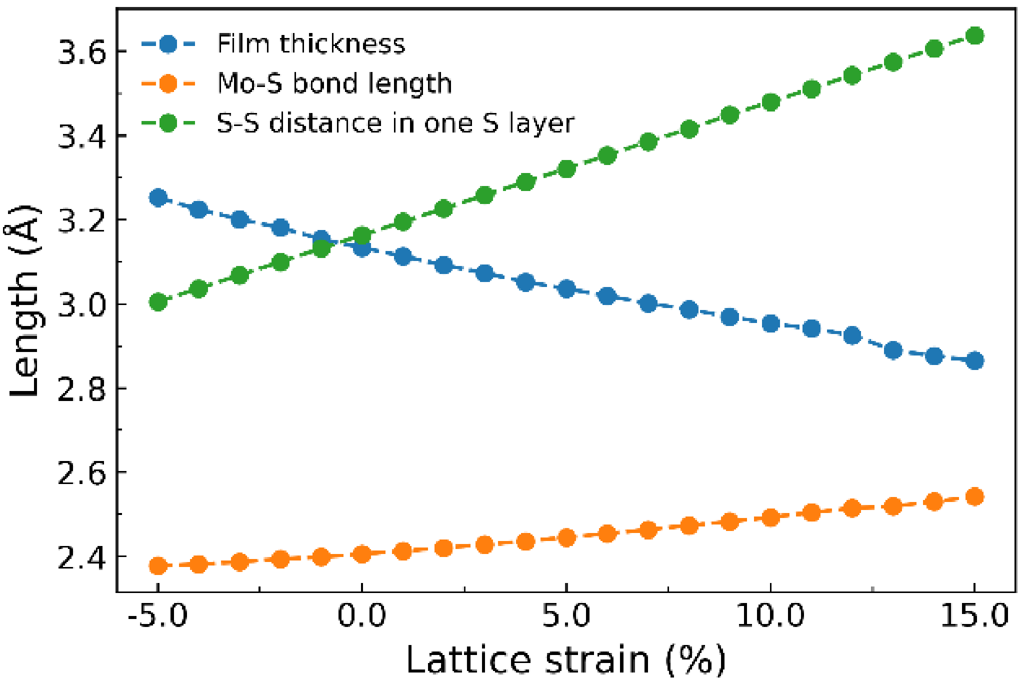

Supplementary Fig. 36. Thickness, Mo−S bond length, and distance between nearest S atoms of the pristine MoS_2 monolayer film at different strain levels. Al the Mo–S bonds are identical in the pristine MoS_2 film. Thickness is defined as the distance between two S layers of a MoS_2 film.

Note: As the lattice strain increased from negative to positive, the Mo–S bonds in the strained film became longer, while two S layers tended to stay closer to the Mo layer to compensate for this elongation, which decreased the film thickness (**Figure S28**).

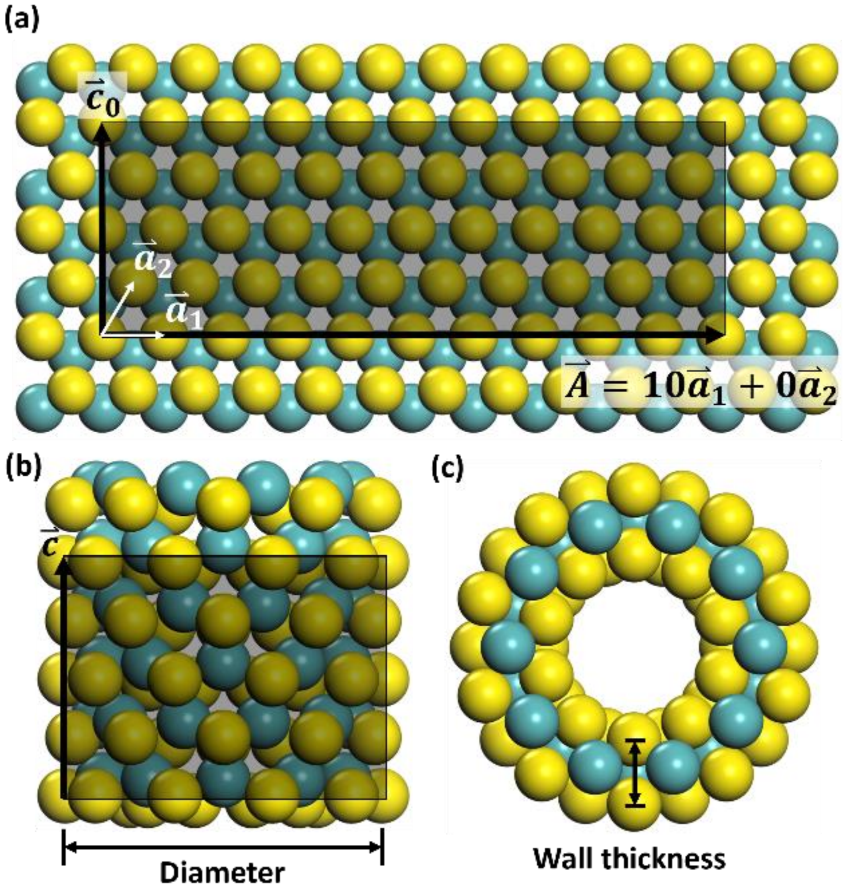

Supplementary Fig. 37. Creation of a (10, 0) zigzag MoS_2 nanotube. (a) Projection (shadow part) of a (10,0) zigzag nanotube unit cell on a MoS_2 film. $\vec{A}$ rolls up to be the perimeter of the nanotube. $\vec{c}_0$ is perpendicular to $\vec{A}$. (b) Side and (c) top view of the (10,0) zigzag nanotube. Each nanotube unit cell consists of four S rings and four Mo rings along $\vec{c}$. S vacancies can form at both the interior and exterior S surfaces.

Note: We created nanotubes with different diameters, namely, (10,0), (12,0), (15,0), (20,0), (25,0) and (30,0) zigzag MoS_2 nanotubes, with construction procedure of the (10,0) nanotube illustrated in **Figure S29**. Diameters, S−S bond lengths, and the strain calculated from the S−S bong length are listed in **Table S4**.

3. Supplementary Tables 1 to 9

Supplementary Table 1. ICP-OES result (wt.%) of elemental composition of catalytic samples

Sample names	Cu content (wt.%)	Zn content (wt.%)	Mo content (wt.%)	MoS_2 content (wt.%)
Commercial $Cu/ZnO/Al_2O_3$ (Alfa Aesar)	60.1	24.8	-	
FL-MoS_2 (Nature Catal., 2021, 4, 242)	-	-	59.9	100
MoS_2@SiO_2	-	-	20.8	34.7
Cu/MoS_2@SiO_2	1.45	-	20.4	34.0

Supplementary Table 2. Structural parameters obtained by EXAFS fitting for catalytic samples and reference materials.

**No.**	**Sample**	**Bond**	**R (Å)[a]**	**CN[b]**	**σ^2(Å^2)[c]**
1	Cu foil	Cu–O (o)	-	-	-
		Cu–S	-	-	-
		Cu–Cu (m)	2.54	12.0*	0.008
2	CuS	Cu–O (o)	-	-	-
		Cu–S	2.25±0.04	3.6*	0.011
		Cu–Cu (m)	-	-	-
3	CuO	Cu–O (o)	1.90±0.05	4*	0.004
		Cu–S	-	-	-
		Cu–Cu (m)			
4	Calcined Cu/MoS_2@SiO_2	Cu–O (o)	1.95±0.00	2.5	0.005
		Cu–S	2.63±0.33	0.6	0.005
		Cu–Cu (m)	-	-	-
5	Reduced Cu/MoS_2@SiO_2	Cu–O (o)	-	-	-
		Cu–S	2.22±0.07	1.5	0.008
		Cu–Cu (m)	2.54±0.01	0.7	0.005

[a] Bond distance in Angstroms; [b] coordination number; [c] Debye-Waller factor.
* These values were fixed during EXAFS fitting, based on known structure of CuS and CuO.
Note: (m) stands for metallic shell, (o) stands for oxide shell.

Supplementary Table 3. Linear combination fitting (LCF) results for reduced $Cu/MoS_2@SiO_2$.

No.	Sample	Standard Weight			R-factor
		CuS	Cu foil	CuO	
1	Calcined	0.00	0.00	1.00	0.0010
2	Reduced	0.931	0.069	0.00	0.0024

Supplementary Table 4. Catalytic performance of different samples in CO_2 hydrogenation to methanol.

Sample names	Reaction conditions	Conversion (%)	MeOH Selectivity (%)	CO Selectivity (%)	CH_4 Selectivity (%)
$Cu@SiO_2$	260 °C, 5 MPa, GHSV=8000 mL·$g_{cat.}^{-1}$·h^{-1}	2.5	50.4	49.6	-
$Cu/MoS_2@SiO_2$		12.8	59.2	23.9	16.4
MoS_2-R		8.9	44.7	24.8	30.5
$MoS_2@SiO_2$		11.1	52.2	22.5	24.6
$MoS_2@SiO_2$-66nm		7.5	50.2	23.6	26.2

MoS_2-R: The SiO_2 shell of $MoS_2@SiO_2$ was removed.

$MoS_2@SiO_2$-66nm: MoO_2 with an average diameter of 66 nm as Mo source.

Supplementary Table 5. The comparison of catalytic performance between the $MoS_2@SiO_2$ and $Cu/MoS_2@SiO_2$ and previous reported Mo-based catalysts.

Catalysts	Reaction conditions	CO_2 Conv. (%)	MeOH Selec. (%)	STY_{MeOH} ($g_{MeOH}\cdot g_{MoS_2}^{-1}\cdot h^{-1}$)	Specific MeOH yield ($mol_{MeOH}\cdot mol_{Mo, Cu\ or\ Pd}^{-1}\cdot h^{-1}$)	References
FL-MoS_2 [a]	180 °C, 5 Mpa, 3000 $mL\cdot g_{cat.}^{-1}\cdot h^{-1}$	9.95	71.7	0.07	0.37	1
FL-MoS_2	240 °C, 5 Mpa, 15000 $mL\cdot g_{cat.}^{-1}\cdot h^{-1}$	11.0	81.4	0.49	2.45	1
FL-MoS_2	260 °C, 5 Mpa, 15000 $mL\cdot g_{cat.}^{-1}\cdot h^{-1}$	15.3	70.1	0.6	3.00	1
FL-MoS_2	260 °C, 5 Mpa, 24000 $mL\cdot g_{cat.}^{-1}\cdot h^{-1}$	12.3	71.0	0.77	3.85	1
Mo_2CT_x/SiO_2	230 °C, 2.5 Mpa,-	-	37	-		2
$Cu/Mo_2CT_x/SiO_2$	230 °C, 2.5 Mpa,-	-	52	-		2
$MoS_2@SiO_2$	230 °C, 2.5 Mpa, 12000 $mL\cdot g_{cat.}^{-1}\cdot h^{-}$	3.14	62.0	0.20	1.00	This work
$Cu/MoS_2@SiO_2$	230 °C, 2.5 Mpa, 12000 $mL\cdot g_{cat.}^{-1}\cdot h^{-}$	3.67	66.5	0.25	1.26	This work
Pd-Cu/Zn	270 °C, 4.5 Mpa, 10800 $mL\cdot g_{cat.}^{-1}\cdot h^{-}$	8.3	64	-	0.89	3
CuO-ZnO-ZrO_2	260 °C, 5 Mpa, 10000 $mL\cdot g_{cat.}^{-1}\cdot h^{-1}$	15.1	42	-	2.87	4
LDHCuZnGa	270 °C, 4.5 Mpa, 18000 $mL\cdot g_{cat.}^{-1}\cdot h^{-}$	18.6	47.5	-	3.73	5
$Cu/ZnO/Al_2O_3$	250 °C, 5 Mpa, 18000 $mL\cdot g_{cat.}^{-1}\cdot h^{-1}$	11.1	54.8	-	1.91	6
$MoS_2@SiO_2$ [a]	180 °C, 5 Mpa, 3000 $mL\cdot g_{cat.}^{-1}\cdot h^{-1}$	4.63	89.3	0.10	0.49	This work
$MoS_2@SiO_2$ [b]	260 °C, 5 Mpa, 24000 $mL\cdot g_{cat.}^{-1}\cdot h^{-1}$	7.46	69.6	0.96	4.80	This work
$MoS_2@SiO_2$ [c]	260 °C, 5 Mpa, 24000 $mL\cdot g_{cat.}^{-1}\cdot h^{-1}$	7.84	72.11	1.08	5.38	This work
$Cu/MoS_2@SiO_{2\ b}$	260 °C, 5 Mpa, 24000 $mL\cdot g_{cat.}^{-1}\cdot h^{-1}$	9.01	72.5	1.22	6.11	This work

[a] Data obtained at the beginning of the long-term stability test.

[b] Data obtained after 150 h of the long-term stability test.

[c] Data obtained after 200 h of the long-term stability test.

Supplementary Table 6. Binding energies of Cu single atom on different sites of MoS_2 basal plane.

Binding site of Cu	[a]E_b / eV	Cu−S bond length / Å	Cu−S coordination
Mo atop	−1.75	2.247	3
Hollow	−1.63	2.305	3
Bridge [b]	−1.50	2.233	2
S atop [b]	−1.17	2.142	1

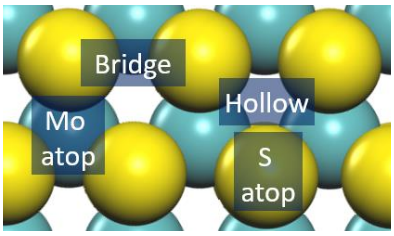

[a] Binding energy E_b is defined as $E_b = E_{Cu/MoS_2} - E_{MoS_2} - E_{Cu}$, where E_{Cu/MoS_2}, E_{MoS_2}, and E_{Cu} are the total energies of Cu-supported MoS_2, pure MoS_2, and single Cu atom.

[b] Binding of Cu to these sites is dynamically unstable because of the presence of imaginary vibrational modes.

Supplementary Table 7. Diameters, S−S bond lengths, and the strain calculated from S−S bond lengths of MoS_2 nanotubes.

Chiral indices	Diameter / nm	Interior S−S bond length / Å	Interior strain / %	Exterior S−S bond length / Å	Exterior strain / %
(10,0)	1.442	2.830	−10.52	3.873	22.45
(12,0)	1.626	2.856	−9.70	3.735	18.08
(15,0)	1.914	2.902	−8.25	3.611	14.16
(20,0)	2.498	2.951	−6.72	3.486	10.21
(25,0)	2.880	2.978	−5.85	3.409	7.75
(30,0)	3.371	3.007	−4.94	3.366	6.41

Supplementary Table 8. Energy decomposition analysis for O* and TS2*.

		E_b / eV [a]	E_d / eV [b]	E_{coads} / eV	E_{int} / eV
O*	-2% strain	−7.26	0.12	0	−7.38
	No strain	−7.36	0.10	0	−7.46
	2% strain	−7.39	0.12	0	−7.52
TS2*	-2% strain	−4.75	0.57	−2.72	−2.60
	No strain	−5.12	0.64	−3.34	−2.43
	2% strain	−5.44	0.36	−3.83	−1.97

[a] Bind energy of (O)* is defined as $E_b = E(int/film) - E(O) - E(film)$, and the binding energy of (TS2)* is defined as $E_b = E(int/film) - E(O) - E(OH) - E(film)$. $E(int/film)$, $E(film)$, $E(O)$, and $E(OH)$ are the total energies of the film with O or TS2 on it, clean MoS_2 film, isolated O, and isolated OH.

[b] Deformation energy E_d comes exclusively from the deformed MoS_2 film, as O doesn't deform, and the contribution from OH deformation is less than 0.002 eV.

Note: To shed light on the unusual increase in the energetic span of O removal when the binding is weaker, we further performed energy decomposition analysis for O* and (TS2)*.[13] The binding energy (E_b) of reaction intermediates and transition states to the film can be decomposed into deformation energy (E_d), adsorbate-adsorbate interaction energy (E_{coads}), and film-intermediate interaction energy (E_{int}), that is, $E_b = E_d + E_{coads} + E_{int}$. E_d is the energy required to deform the adsorbates and the film to their geometries in the combined structure from their separately obtained equilibrium structures. E_{coads} is the change in energy for bringing the deformed adsorbates together to form the intermediate. E_{int} is the energy change when bringing the intermediate and the deformed film together. Definitions of the binding energy for (O)* and (TS2)* and the values of each term are shown in **Supplementary Table 8**. Since Gibbs energy corrections for (O)* and (TS2)* after applying strain varied by less than 0.013eV, the change in the energetic span after applying strain can be approximated as the following: $\Delta(\delta E) \approx \Delta E_b(TS2) - \Delta E_b(O) = \Delta E_d(TS2) - \Delta E_d(O) + \Delta E_{coads}(TS2) - \Delta E_{coads}(O) + \Delta E_{int}(TS2) - \Delta E_{int}(O)$. As O is small, compressively strained film did not need to deform more for the O to reside, resulting in a small change in E_d after applying 2% compressive strain ($\Delta E_d(O) = 0.02$ eV). For TS2, as suggested by $\Delta E_d(TS2) = -0.07$ eV, the film was less deformed for adsorbing TS2 when 2% compressive strain was applied. E_{coads} for (O)* is always zero because O does not interact with other adsorbates. And $\Delta E_{coads}(TS2)$ is 0.62 eV with 2% compressive strain, indicating a much weaker interaction between H and OH, which can be explained by the longer H-OH distance on that strained film (**Supplementary Table 9**). The interaction between O and the deformed film is weaker with 2% compressive strain ($\Delta E_{int}(O) = 0.08$ eV). However, $\Delta E_{int}(TS2)$ is -0.17 eV after 2% compressive strain applying, showing an enhanced interaction between TS2 and the deformed film. This enhanced interaction can be explained by the larger deviation of TS2 from H_2O molecule, which is suggested by the longer H-OH distance, larger H-O-H angle, and less positive charge of the attacking H (**Supplementary Table 9**). As H_2O is weakly bound to the film compared to (OH+H)*, this larger deviation results in stronger interaction between TS2 and the film. Overall, due to the most significant contribution from $\Delta E_{coads}(TS2)$, the energetic span increased after applying 2% compressive strain ($\Delta(\delta E) = 0.28$ eV). 2% tensile strain has the opposite effects and decreased the energetic span for O hydrogenation by 0.27 eV.

Supplementary Table 9. Structural parameters and charges of reaction intermediates from O* to TS2*.

Reaction intermediates	Strain	Charges of O / |e|	Charges of first attacking H / |e|	Charges of second attacking H / |e|	H−OH bond length / Å	H−O−H bond angle
O*	No strain	-0.98	-	-	-	-
	-2% strain	-0.97	-	-	-	-
	2% strain	-0.99	-	-	-	-
(O+H+H)*	No strain	-0.99	-0.20	-0.29	-	-
	-2% strain	-0.99	-0.24	-0.25	-	-
	2% strain	-0.99	-0.19	-0.30	-	-
(OH+H)*	No strain	-1.26	0.71	-0.34	-	-
	-2% strain	-1.30	0.76	-0.34	-	-
	2% strain	-1.25	0.70	-0.34	-	-
TS2*	No strain	-1.20	0.66	0.20	1.326	131.0°
	-2% strain	-1.20	0.66	0.15	1.387	138.6°
	2% strain	-1.23	0.69	0.24	1.264	126.9°
H_2O*	No strain	-1.33	0.68	0.66	0.983	106.6°
	-2% strain	-1.31	0.67	0.65	0.983	106.3°
	2% strain	-1.36	0.70	0.68	0.983	106.9°

4. Supplementary References

1. Hu, J. et al. Sulfur vacancy-rich MoS_2 as a catalyst for the hydrogenation of CO_2 to methanol. *Nat. Catal.* **4**, 242-250 (2021).
2. Zhou, H. et al. Engineering the Cu/Mo_2CT_x (MXene) interface to drive CO_2 hydrogenation to methanol. *Nat. Catal.* **4**, 860-871 (2021).
3. Hu, B. et al. Hydrogen spillover enabled active Cu sites for methanol synthesis from CO_2 hydrogenation over Pd doped CuZn catalysts. *J. Catal.* **359**, 17-26 (2018).
4. Angelo, L. et al. Catalyst synthesis by continuous coprecipitation under micro-fluidic conditions: Application to the preparation of catalysts for methanol synthesis from CO_2/H_2. *Catal. Today* **270**, 59-67 (2016).
5. Li, M.M.J. et al. CO_2 hydrogenation to methanol over catalysts derived from single cationic layer CuZnGa LDH precursors. *ACS Catal.* **8**, 4390-4401 (2018).
6. An, B. et al. Confinement of ultrasmall Cu/ZnO_x nanoparticles in metal–organic frameworks for selective methanol synthesis from catalytic hydrogenation of CO_2. *J. Am. Chem. Soc.* **139**, 3834-3840 (2017).
